# Prediction of differentially expressed microRNAs in blood as potential biomarkers for Alzheimer’s disease by meta-analysis and adaptive boosting ensemble learning

**DOI:** 10.1186/s13195-021-00862-z

**Published:** 2021-07-09

**Authors:** Sze Chung Yuen, Xiaonan Liang, Hongmei Zhu, Yongliang Jia, Siu-wai Leung

**Affiliations:** 1 State Key Laboratory of Quality Research in Chinese Medicine, Institute of Chinese Medical Sciences, University of Macau, Avenida da Universidade, Taipa, 999078 Macao China; 2 BGI College & Henan Institute of Medical and Pharmaceutical Sciences, Zhengzhou University, Zhengzhou, Henan China; 3 Department of Obstetrics and Gynecology, The Second Affiliated Hospital of Zhengzhou University, Zhengzhou, Henan China; 4 Shenzhen Institute of Artificial Intelligence and Robotics for Society, Shenzhen, China; 5 Edinburgh Bayes Centre for AI Research in Shenzhen, College of Science and Engineering, University of Edinburgh, Edinburgh, Scotland, UK

**Keywords:** Alzheimer’s disease, Meta-analysis, MicroRNAs, ABMDA, Biomarkers, Neuroinflammation, Neuronal cell cycle re-entry

## Abstract

**Background:**

Blood circulating microRNAs that are specific for Alzheimer’s disease (AD) can be identified from differentially expressed microRNAs (DEmiRNAs). However, non-reproducible and inconsistent reports of DEmiRNAs hinder biomarker development. The most reliable DEmiRNAs can be identified by meta-analysis. To enrich the pool of DEmiRNAs for potential AD biomarkers, we used a machine learning method called adaptive boosting for miRNA disease association (ABMDA) to identify eligible candidates that share similar characteristics with the DEmiRNAs identified from meta-analysis. This study aimed to identify blood circulating DEmiRNAs as potential AD biomarkers by augmenting meta-analysis with the ABMDA ensemble learning method.

**Methods:**

Studies on DEmiRNAs and their dysregulation states were corroborated with one another by meta-analysis based on a random-effects model. DEmiRNAs identified by meta-analysis were collected as positive examples of miRNA–AD pairs for ABMDA ensemble learning. ABMDA identified similar DEmiRNAs according to a set of predefined criteria. The biological significance of all resulting DEmiRNAs was determined by their target genes according to pathway enrichment analyses. The target genes common to both meta-analysis- and ABMDA-identified DEmiRNAs were collected to construct a network to investigate their biological functions.

**Results:**

A systematic database search found 7841 studies for an extensive meta-analysis, covering 54 independent comparisons of 47 differential miRNA expression studies, and identified 18 reliable DEmiRNAs. ABMDA ensemble learning was conducted based on the meta-analysis results and the Human MicroRNA Disease Database, which identified 10 additional AD-related DEmiRNAs. These 28 DEmiRNAs and their dysregulated pathways were related to neuroinflammation. The dysregulated pathway related to neuronal cell cycle re-entry (CCR) was the only statistically significant pathway of the ABMDA-identified DEmiRNAs. In the biological network constructed from 1865 common target genes of the identified DEmiRNAs, the multiple core ubiquitin-proteasome system, that is involved in neuroinflammation and CCR, was highly connected.

**Conclusion:**

This study identified 28 DEmiRNAs as potential AD biomarkers in blood, by meta-analysis and ABMDA ensemble learning in tandem. The DEmiRNAs identified by meta-analysis and ABMDA were significantly related to neuroinflammation, and the ABMDA-identified DEmiRNAs were related to neuronal CCR.

**Supplementary Information:**

The online version contains supplementary material available at 10.1186/s13195-021-00862-z.

## Background

Alzheimer’s disease (AD) is subcellularly characterized by the presence of extracellular amyloid-beta (Aβ) plaque deposition and intracellular neurofibrillary tangles of hyperphosphorylated tau proteins [1]. The aberrant protein aggregates are accompanied by activation of neuroinflammation, and loss of synaptic functions [2]. In the progression of AD, irreversible loss of neurons and synaptic functions gradually develops over decades before the manifestation of cognitive symptoms [3]. Because the root causes of pathological Aβ accumulation and hyperphosphorylated tau proteins are not clear, drug development for AD often fails and current AD treatments alleviate symptoms only. The failure of most clinical trials in AD has been partially attributed to the lack of sensitive biomarkers to identify potential AD [4], which can identify and enroll patients at the early stage of AD, as it may be too late to rescue the dysfunction present in advanced stages of the disease.

The National Institute of Neurological and Communicative Disorders and Stroke and the Alzheimer’s Disease and Related Disorders Association (NINCDS-ADRDA) and the National Institute on Aging and Alzheimer’s Association (NIA-AA) proposed that the diagnosis of AD should be dependent on biomarkers rather than solely dependent on clinical symptoms [5, 6]. Aberrant levels of Aβ and tau proteins in cerebrospinal fluid (CSF) and blood have been evaluated as biomarkers for AD diagnosis [7, 8], specifically, increased levels of total and hyperphosphorylated tau proteins, and decreased levels of Aβ in CSF. CSF levels of total and hyperphosphorylated tau proteins are correlated with neurofibrillary tangle load, and CSF levels of Aβ are inversely correlated with amyloid load [9]. However, a review concluded that these CSF biomarkers are more useful for ruling out AD, than for indicating a definite diagnosis [10]. To avoid the side effects of invasive CSF sampling, such as positional headache, Fei et al. [11] have proposed the use of peripheral blood Aβ detection, such as the ratio of Aβ42/Aβ40. However, another review has reported that lower plasma Aβ42/Aβ40 ratios might not be associated with increased AD risk [12]. The plasma phosphorylated tau 217 is proposed to distinguish AD from non-AD neurodegenerative individuals, since its level increases more steeply in non-demented individuals with amyloid positivity than those without amyloid positivity [13]. The limitations to use Aβ or tau proteins as biomarkers in blood might be due to several reasons. First, Aβ in both CSF and blood tends to self-aggregate [14], masking epitopes for detection and reducing the correlation with AD. Second, the entrance of Aβ and tau proteins into body fluid is hindered by the extent of blood–brain barrier (BBB) leakage [15]. Thus, there is a need to identify other potential non-invasive biomarkers to aid the diagnosis of AD. An ideal AD biomarker would allow for mass screening to identify patients at high risk of developing AD in the presymptomatic stage with adequate reliability.

MicroRNAs (miRNAs) are a class of single-stranded non-coding RNAs that are 18–25 nucleotides in length and bind to the 3′ untranslated region of target mRNAs to modify the target mRNAs’ expression in a post-transcriptional manner [16]. Each miRNA simultaneously targets hundreds of mRNAs, and over 2500 mature miRNAs have been recorded in the latest version of the miRBase database [17]. Out of all recorded miRNAs, 300 have been associated with neurodegenerative diseases and 131 miRNAs are specific for AD [18]. Downregulated expressions of miR-29a/b-1, miR-29c, and miR-339-5p have been reported to upregulate the expression of *BACE1* in AD brain, thereby increasing Aβ production [19–21]. The expression of miR-15/107 in cerebral cortical gray matter is correlated with amyloid plaque density [22]. The AD-dysregulated miRNAs in brain have been associated with neuroinflammation and cell cycle regulation [23, 24] and may be released into peripheral blood through the BBB [25] and transported by lipoproteins in circulation for stability [26]. These stable AD-dysregulated miRNAs in blood may reflect the composition of dysregulated miRNAs in brain, suggesting that they may be potential biomarkers of AD. The dysregulated miRNAs were found in plasma and serum of AD patients [27, 28], e.g., the expression of miR-125b in serum of AD patients is correlated with Mini-Mental State Examination (MMSE) scores [29]. Compared with biomarkers in brain or CSF, blood circulating biomarkers are preferable for their higher accessibility. However, before establishing any miRNAs as AD biomarkers, it is necessary to first evaluate their reliability and consistency among different studies of differentially expressed miRNAs (DEmiRNAs) in AD patients, and their biological significance to understand their roles in the pathogenesis of AD.

Thus far, the development of blood circulating DEmiRNAs as AD biomarkers has been hindered by inconsistent and unreliable studies. For example, Denk et al. [30] and Wu et al. [31] conducted similar studies using quantitative real-time polymerase chain reaction (qRT-PCR) to quantify the expression of miRNAs in serum, and found 22 and 9 DEmiRNAs, respectively. Only one DEmiRNA, miR-146a-5p, was common between the two studies. Further, the studies reported the same miR-146a-5p to have opposite directions of dysregulation in AD. This inconsistency can be attributed to differences in the AD patients recruited for the trials, including the presence of other disease conditions that might influence the levels of biological molecules in blood, and the stages of disease progression [32, 33]. A meta-analysis of these inconsistent DEmiRNA results may resolve discrepancies and enhance generalizability of the results. Takousis et al. [34] conducted a meta-analysis to identify DEmiRNAs in brain, blood, and CSF from expression profiling studies of AD. They applied Stouffer’s method to integrate the *P* values of every DEmiRNA from each independent study and found 32 statistically significant DEmiRNAs in blood. However, Stouffer’s method does not include information about dysregulation states of DEmiRNAs and its use of *P* values is outdated in terms of the methodology of meta-analysis; thus, the results were probably biased by the number of studies reporting the same DEmiRNAs. Hu et al. and Zhang et al. [35, 36] also conducted meta-analyses independently and reported that the sensitivity (86% and 80%, respectively) and specificity (87% and 83%, respectively) of blood-based miRNAs are comparable with those of fluorodeoxyglucose-positron emission tomography (FDG-PET; sensitivity: 91%, specificity: 86%) [37] for AD diagnosis. The results of these meta-analyses demonstrate that some blood circulating DEmiRNAs may be useful for AD diagnosis, but there has not been extensive identification of DEmiRNAs that may serve as AD biomarkers.

Structural and functional patterns of similar DEmiRNAs can be identified by machine learning, which has been applied to the identification of AD biomarkers. Studies using different machine learning techniques have identified different DEmiRNAs for AD diagnosis with up to 89% accuracy [38–40]. Until recently, machine learning mainly focused on fine tuning the set of DEmiRNAs identified from differential miRNA expression profiling studies to obtain a smaller panel of miRNAs for AD diagnosis. As an emerging trend, machine learning is being used to predict potential DEmiRNAs as AD biomarkers based on DEmiRNAs identified from differential expression profiling studies. For example, Zhao et al. [41] used the adaptive boosting for miRNA disease association (ABMDA) ensemble learning method to identify miRNAs that are associated with a disease. ABMDA is a supervised learning approach with validation against the Human MicroRNA Disease Database (HMDD) [42]. Its clustering algorithm is based on *k*-means distance, and its boosting technique combines classifiers by their corresponding weights to form a stronger classifier. The ABMDA ensemble learning method relies upon the initial set of DEmiRNAs to predict additional DEmiRNAs. In the present study, we used meta-analysis to enhance the reliability of the initial DEmiRNAs and augment the prediction performance of the ABMDA ensemble learning method.

This study aimed to identify blood circulating DEmiRNAs as potential AD biomarkers by augmenting the DEmiRNAs identified by meta-analysis with the ABMDA ensemble learning method. The meta-analysis was conducted under the guidance of the PRISMA statement [43], with the adjustment in the risk of bias assessment.

## Methods

**Fig. 1:**
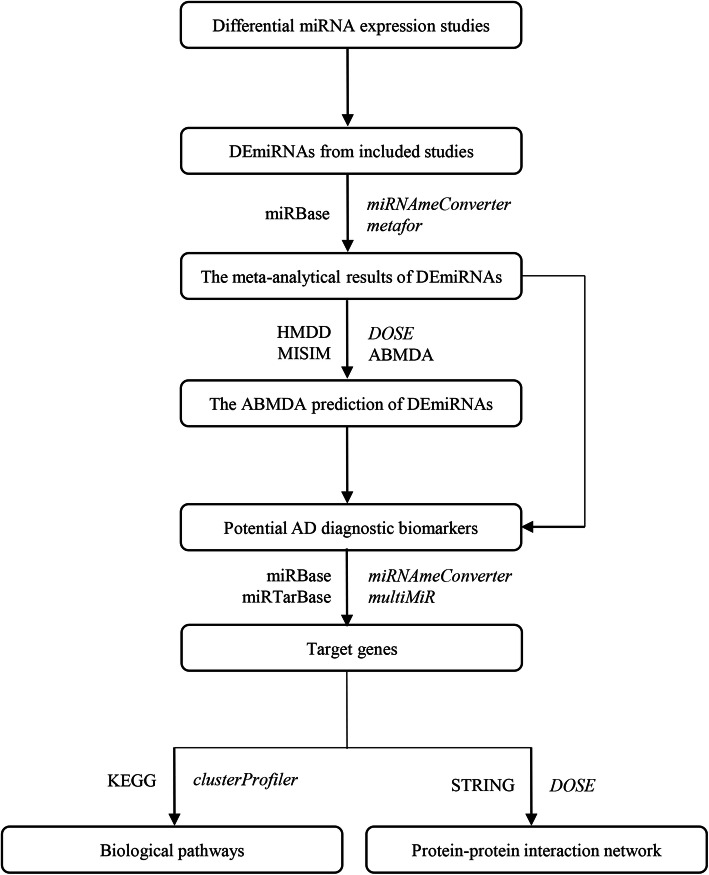
Overall study design. The italic font and normal font on the right-hand side of the arrows represent the R package and python-based algorithm, respectively. The name of each database is shown on the left-hand side of the arrow

The present meta-analysis was mainly conducted using R and Python packages and is illustrated in Fig. 1. If an included study contained several comparisons, the DEmiRNAs from each comparison were collected independently. The sample sizes of control and AD groups, and DEmiRNAs and their dysregulated states from each independent comparison were extracted. The names of all reported DEmiRNAs were standardized based on the miRBase database [17] before the meta-analysis. A meta-analysis for the DEmiRNAs was performed when more than one comparison reported the same DEmiRNA. Statistically significant DEmiRNAs from the meta-analysis were collected as positive miRNA–AD associations for the ABMDA ensemble learning method to further increase the number of positive miRNA–AD associations. The ABMDA-identified miRNAs with scores higher than the predefined criteria, together with the DEmiRNAs identified by the meta-analysis, were treated as potential AD biomarkers in blood. The biological significance of the identified DEmiRNAs was studied by a biological pathway enrichment analysis and network analysis of their corresponding target genes. The combination of meta-analysis and ABMDA ensemble learning might be beneficial to resolve the existing inconsistencies and perform systematical predictions based on reliable results.

### Differential miRNA expression studies selection

Differential miRNA expression studies were collected from PubMed from inception until January 10, 2020, from ScienceDirect and Web of Science between 1993 and 2019. The search strategy is shown below:

For PubMed, (“Alzheimer’s disease” [MESH Terms] OR “Alzheimer’s disease” [All Fields] OR “alzheimer*” [MESH Terms] OR “alzheimer*” [All Fields]) AND (“microRNA*” [MESH Terms] OR “microRNA*” [All Fields] OR “miRNA*” [MESH Terms] OR “miRNA*” [All Fields])

For ScienceDirect and Web of Science, (microRNA OR miRNA) AND Alzheimer

After the search, two authors (SCY and XNL) screened all titles, abstracts, and full texts independently according to the eligibility criteria. Disagreements between the authors were resolved by discussion with the other authors.

The case-control studies reporting differential miRNA expression in blood from AD and healthy participants were included. The included studies had to report the differential expression profiling methods, sample sizes of the disease and control groups, and statistical significance for each DEmiRNA. Studies were excluded if they (1) were not AD research (i.e., focused on other dementia disease conditions); (2) reported irrelevant effects on differential miRNA expression profiles (i.e., demonstrated treatment effects on the differential miRNA expression); (3) did not report the blood DEmiRNAs for AD and healthy participants (i.e., were not controlled studies, or recruited AD participants suffering from other diseases); (4) did not report DEmiRNAs and their corresponding dysregulation states (i.e., up or down) as outcomes; or (5) did not obtain DEmiRNAs by a validation method (i.e., qRT-PCR).

### Meta-analysis

For each study, the following data were extracted for the meta-analysis: (1) comparison ID (PubMed ID with blood elements or disease stages); (2) blood elements; (3) differential miRNA expression profiling method; (4) number of AD cases; (5) number of control cases; and (6) DEmiRNAs and their dysregulated states. For studies that reported both screening and validation results of differential miRNA expression, only the results from validation methods were collected. The names of extracted DEmiRNAs were standardized using the *miRNAmeConverter* [44] package for R software with the miRBase database [17]. For each DEmiRNA that was reported by more than one comparison, a meta-analysis was performed using the *metafor* [45] package for R software. The meta-analysis was conducted for each qualified DEmiRNA, independently, under a random-effects model. For each qualified DEmiRNA from independent comparisons, that was reported as binary dysregulation (i.e., upregulation or downregulation) in AD group compared with healthy control group. The effect sizes of binary data were calculated as log_e_ odds ratios (logORs) based on the number of dysregulation events in both disease and control samples. The heterogeneity of each DEmiRNA was reported as tau square (τ^2^) based on the restricted maximum-likelihood estimator and *I*^2^ statistics. The outcomes were logORs with a 95% confidence interval (CI), *P* values, τ^2^, and *I*^2^. For each DEmiRNA in the *i*th study, the effect size (*θ*_*i*_ ) based on the numbers of dysregulation events in both AD and control samples was calculated according to the formula, log($\frac{A_i\ast {D}_i}{B_i\ast {C}_i}$), where A_*i*_ and B_*i*_ (C_*i*_ and D_*i*_) represent the number of upregulated and downregulated cases in the disease (control) group, respectively. Then the overall effect was computed according to formula, $\frac{\sum {W}_i{\theta}_i}{\sum {W}_i}$, where W_*i*_ is the weight and is equal to 1/(v_*i*_ + τ^2^), where v_*i*_ is the sample variance. A larger sample size has more weight on the overall effect size. The *P* values were adjusted by false discovery rate (FDR), and DEmiRNAs with FDR-adjusted *P* values less than 0.05 were regarded as statistically significant. Statistically significant DEmiRNAs with logORs above or below 0 were considered upregulated or downregulated, respectively, in AD compared with healthy controls.

Subgroup analysis was conducted based on the DEmiRNAs collected from the included differential miRNA expression studies. The DEmiRNAs were split into four different subgroups based on the blood sample sources, i.e., whole blood, plasma, serum, and peripheral blood mononuclear cell (PBMC).

### ABMDA identification of DEmiRNAs

ABMDA ensemble learning was used to identify potential miRNA–disease associations. The original miRNA–disease associations with significant dysregulation in the “circulation_biomarker_diagnosis” category were extracted from HMDD, in which the associations are experimentally verified. DEmiRNAs identified in the meta-analysis with adjusted *P* values less than 0.05 were input into the ABMDA ensemble learning method as positive miRNA–AD associations to further increase the number of positive miRNA–AD associations. In addition to initial miRNA–disease associations, ABMDA ensemble learning also requires disease–disease similarity and miRNA–miRNA functional similarity. The disease–disease similarity was determined using the *DOSE* [46] package for R software. The miRNA–miRNA functional similarity was retrieved from the database MISIM [47]. The ABMDA-identified DEmiRNAs were sorted by their prediction scores, and the DEmiRNAs with top prediction scores were collected until the first predicted DEmiRNA that was reported statistically insignificant in the meta-analysis. The names of predicted DEmiRNAs were standardized using the *miRNAmeConverter* package for R software with the miRBase database [17]. The ABMDA-identified DEmiRNAs with the top prediction scores, together with the meta-analysis DEmiRNAs were considered potential AD biomarkers.

### Biological significance of DEmiRNAs from meta-analysis and ABMDA by independent and synergic biological enrichment analysis

Each miRNA regulates a large number of genes to exert a profound influence on genetic expression in specific cellular functions, and the primary function of each miRNA can be understood by identifying its target genes. The target genes of the DEmiRNAs from the meta-analysis and ABMDA identification were obtained using the *multiMiR* [48] package for R software based on the database miRTarBase [49]. The target genes of two DEmiRNA categories were collected separately to conduct two independent enrichment analyses and were also combined to conduct a synergic enrichment analysis. The biological enrichment analysis was conducted using the *clusterProfiler* [50] package for R software based on the Kyoto Encyclopedia of Genes and Genomes (KEGG) [51]. The pathways with FDR-adjusted *P* values less than 0.05 were considered statistically significant.

### Collaborative biological function of DEmiRNAs from meta-analysis and ABMDA by network analysis

The target gene semantic similarity measurements of the DEmiRNAs identified from the meta-analysis and ABMDA were computed using the *DOSE* package for R. The target genes with semantic similarity over 0.95 were treated as common target genes in the two DEmiRNA categories. The DEmiRNAs and common target genes of the two DEmiRNA categories were used to construct a DEmiRNA–gene network based on the data from STRING (version 11) [52] to investigate the collaborative function of the DEmiRNAs identified from the meta-analysis and ABMDA. Only interactions with the highest confidence (0.9) were kept from the STRING.

### Risk of bias

The risk of bias for each included study was evaluated according to the Minimum Information for Publication of Quantitative Real-time PCR Experiments (MIQE) [53]. This guideline is designed to assess the quality of qRT-PCR data and describes the minimum information required to ensure that experimental results can be comprehensively interpreted and independently verified. In the present study, the guideline was used to assess the quality of the expression profiling analysis of the included studies, including experimental design, sample annotation, experimental procedure, data processing pipeline, and result presentation. The evaluation was performed by two authors (SCY and XNL), independently. Disagreements between the authors were resolved by discussion with the other authors. Items with low risk were counted + 1, suggesting high reproducibility; items with unclear risk were counted 0, suggesting ambiguous reproducibility; and items with high risk were counted − 1, suggesting low reproducibility.

## Results

### Differential miRNA expression studies included in the analysis

**Fig. 2:**
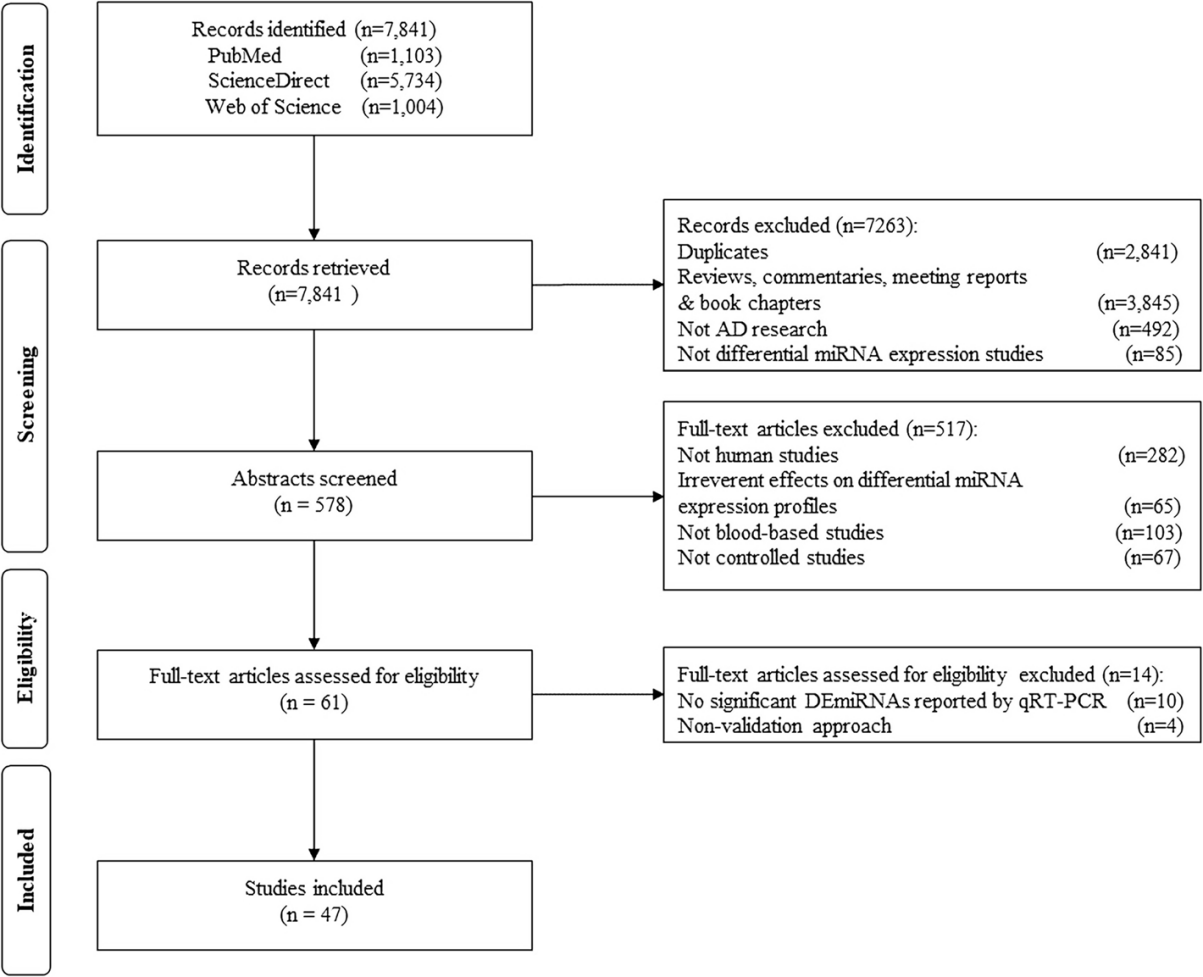
Flow diagram of the differential miRNA expression study selection, including the identification, screening, eligibility, and inclusion stages

The selection process of the differential miRNA expression studies is shown in Fig. 2. A total of 7841 studies were initially identified from PubMed, ScienceDirect, and Web of Science. After a systematic search, 47 studies met the eligibility criteria and were included in the present study [23, 27–31, 39, 40, 54–92]. The characteristics of the included studies are shown in Table 1. The studies mainly focused on four blood elements: serum, plasma, whole blood, and peripheral blood mononuclear cells. Serum (*n* = 19) was the most extensively studied, followed by plasma (*n* = 16).

**Table 1:** Characteristics of the included differential miRNA expression studies

PubMed ID	Comparison ID	Blood elements	Differential miRNA expression profiling methods (screening method/validation method)	Number of control case	Number of AD case	Number of upregulated miRNA	Number of downregulated miRNA
31857133	31857133_Ser	Serum	qRT-PCR	93	108		1
31811079	31811079_Ser	Serum	qRT-PCR	51	32	9	3
31849573	31849573_Pla	Plasma	microRNA microarray/qRT-PCR	31	16		2
31809862	31809862_WB	Whole blood	qRT-PCR	214	145	1	1
31766231	31766231_Pla	Plasma	microRNA microarray/qRT-PCR	29	23	1	
31691877	31691877_Ser	Serum	qRT-PCR	30	30	4	
31592314	31592314_Pla	Plasma	RNA deep-sequencing/qRT-PCR	11	10		6
31572518	31572518_Ser	Serum	qRT-PCR	98	105		1
31420923	31420923_Pla	Plasma	qRT-PCR	120	120		2
31092279	31092279_Pla	Plasma	qRT-PCR	14	56	3	
30914454	30914454_Pla	Plasma	qRT-PCR	385	385		5
30328325	30328325_Pla	Plasma	qRT-PCR	20	20	1	
29966198	29966198_Pla	Plasma	qRT-PCR	10	10	2	
29746584	29746584_Ser	Serum	qRT-PCR	38	47	14	8
29635818	29635818_Pla	Plasma	qRT-PCR	20	20	1	
29606187	29606187_Ser	Serum	qRT-PCR	107	228	2	1
29036829	29036829_WB	Whole blood	microRNA microarray/qRT-PCR	17	21		3
29036818	29036818_Ser_Mild	Serum	RNA deep-sequencing/qRT-PCR	86	31	1	5
	29036818_Ser_Mod	Serum	RNA deep-sequencing/qRT-PCR	86	52	3	5
	29036818_Ser_Sev	Serum	RNA deep-sequencing/qRT-PCR	86	38	4	5
28934394	28934394_Ser	Serum	microRNA microarray/qRT-PCR	18	11	1	
28849039	28849039_Ser_Mil	Serum	microRNA microarray/qRT-PCR	30	30		1
	28849039_Ser_Mod	Serum	microRNA microarray/qRT-PCR	30	30		1
28626163	28626163_Ser	Serum	RNA deep-sequencing/qRT-PCR	40	45	5	4
28179587	28179587_Pla_AD2	Plasma	qRT-PCR	9	13	4	3
	28179587_Pla_MCI-AD2	Plasma	qRT-PCR	9	8	4	
28137310	28137310_Ser	Serum	RNA deep-sequencing/qRT-PCR	22	36		1
27545218	27545218_Ser	Serum	qRT-PCR	40	48	1	
27501295	27501295_WB	Whole blood	qRT-PCR	109	172		4
27446280	27446280_WB	Whole blood	qRT-PCR	25	25		1
27277332	27277332_WB	Whole blood	qRT-PCR	30	30	1	
27239545	27239545_PBMC	Peripheral blood mononuclear cells	qRT-PCR	36	36	3	
27027823	27027823_Ser	Serum	qRT-PCR	62	84	1	3
26973465	26973465_Pla	Plasma	microRNA microarray/qRT-PCR	40	40		3
26497032	26497032_PBMC	Peripheral blood mononuclear cells	microRNA microarray/qRT-PCR	41	45	2	1
26078483	26078483_Ser	Serum	RNA deep-sequencing/qRT-PCR	75	79		4
25955795	25955795_WB	Whole blood	qRT-PCR	30	30		1
25742200	25742200_Pla	Plasma	qRT-PCR	81	97		1
25667669	25667669_Ser	Serum	qRT-PCR	42	26		1
25152461	25152461_Ser	Serum	microRNA microarray/qRT-PCR	30	38		2
24827165	24827165_Pla	Plasma	qRT-PCR	7	7		1
	24827165_Ser	Serum	qRT-PCR	7	7		1
24577456	24577456_Ser	Serum	RNA deep-sequencing/qRT-PCR	155	158		6
24550773	24550773_Pla	Plasma	qRT-PCR	27	25	1	
	24550773_PBMC	Peripheral blood mononuclear cells	qRT-PCR	27	25	1	
24157723	24157723_Pla	Plasma	qRT-PCR	10	10		2
24139697	24139697_Ser	Serum	qRT-PCR	155	105	1	2
24064186	24064186_PBMC_Mon	Monocytes	qRT-PCR	37	34	1	
	24064186_PBMC_Lym	Lymphocytes	qRT-PCR	37	34	1	
23922807	23922807_Pla_C2	Plasma	microRNA microarray/qRT-PCR	17	20		6
23895045	23895045_WB	Whole blood	RNA deep-sequencing/qRT-PCR	21	94	5	7
23435408	23435408_PBMC	Peripheral blood mononuclear cells	qRT-PCR	25	28		1
22155483	22155483_Ser	Serum	qRT-PCR	7	7		5
19936094	19936094_PBMC	Peripheral blood mononuclear cells	microRNA microarray/qRT-PCR	5	5	2	

### Identification of DEmiRNAs from meta-analysis

After DEmiRNA name standardization, there were 115 DEmiRNAs reported in 54 independent comparisons of 47 differential expression studies that compared AD blood samples with healthy blood samples. Eight-eight DEmiRNAs were found in one blood element, 25 DEmiRNAs were found in two blood elements, two DEmiRNAs were found in three blood elements, and no DEmiRNAs were found in all four blood elements. The most frequently reported DEmiRNAs in AD blood were miR-146a-5p and miR-26a-5p, which were dysregulated in five independent comparisons (Table 2). Of the 115 DEmiRNAs, 43 (37.4%) were reported in at least two independent comparisons; dysregulation of 18 of them was reported consistently in the same direction, whereas dysregulation of 25 of them was reported in different directions. Based on the currently available data, none of the inconsistent DEmiRNA results were resolved in this meta-analysis. In the meta-analysis of 43 dysregulated miRNAs, 18 DEmiRNAs were found to be statistically significant (Table 3; Additional file 1); 7 and 11 DEmiRNAs were upregulated and downregulated, respectively. Among the 18 DEmiRNAs identified in this study, 6 of them (let-7d-5p, miR-107, miR-128-3p, miR-191-5p, miR-29c-3p, and miR-93-5p) were also found statistically significant in a previous meta-analysis [34]. The discrepancy could be due to that we only included the literature with qRT-PCR as validation results, and we treated comparisons independently even that those comparisons were from the same literature. Meanwhile, 13 DEmiRNAs of our meta-analytical results (let-7d-5p, miR-106b-3p, miR-107, miR-126-5p, miR-148b-5p, miR-181c-3p, miR-191-5p, miR-200a-3p, miR-22-3p, miR-483-5p, miR-486-5p, miR-502-3p, and miR-93-5p), were reported contributing the AD diagnostic values in sensitivity and specificity in Hu et al. and Zhang et al. [35, 36]. The most significantly downregulated DEmiRNA was miR-107, which was identified in four independent comparisons. MiR-106b-39 was the most significantly upregulated DEmiRNA among four independent comparisons. Downregulation of miR-107 has been reported to increase *BACE1* expression [93] and influence cell cycle protein expression [94]. The dysregulation of miR-106b-3p is negatively correlated with MMSE score [66] and modulates Aβ metabolism [95]. Most DEmiRNAs identified in the meta-analysis were associated with mediating Aβ generation, tau protein phosphorylation, and neuronal functions maintenance.

**Table 2:** DEmiRNAs reported by the included differential miRNA expression studies. The bold DEmiRNAs were reported by at least two independent comparisons and were qualified for the subsequent meta-analysis

Comparison ID	MiRNAs	Dysregulated direction	***P*** value
31857133_Ser	miR-193a-3p	Down	< 0.001
31811079_Ser	miR-346	Up	0.0013
	miR-345-5p	Up	0.0239
	miR-122-3p	Up	0.0001
	miR-1291	Up	0.0052
	miR-640	Up	0.0004
	miR-650	Up	0.0035
	miR-1285-3p	Up	0.0032
	miR-1299	Up	0.0003
	miR-1267	Up	0.0055
	miR-208b-3p	Down	0.0006
	miR-206	Down	0.0004
31849573_Pla	**miR-132-3p**	Down	0.0333
	miR-212-3p	Down	0.001
31809862_WB	miR-532-5p	Up	4.8 × 10E−30
	miR-1468-5p	Down	6.2 × 10E−12
31766231_Pla	miR-206	Up	< 0.025
31691877_Ser	miR-22-5p	Up	≤ 0.005
	miR-23a-3p	Up	≤ 0.05
	miR-29a-3p	Up	≤ 0.05
	miR-125b-5p	Up	≤ 0.005
31592314_Pla	miR-451a	Down	< 0.0005
	miR-21-5p	Down	< 0.005
	miR-23a-3p	Down	< 0.005
	let-7i-5p	Down	< 0.05
	miR-126-3p	Down	< 0.005
	miR-151a-3p	Down	< 0.05
31572518_Ser	miR-133b	Down	< 0.001
31420923_Pla	miR-103a-3p	Down	< 0.001
	**miR-107**	Down	< 0.001
31092279_Pla	miR-92a-3p	Up	0.0442
	miR-181c-5p	Up	0.0024
	miR-210-3p	Up	0.0006
30914454_Pla	miR-101-3p	Down	< 0.001
	miR-153-3p	Down	< 0.001
	miR-144-3p	Down	< 0.001
	miR-381-3p	Down	< 0.001
	miR-383-5p	Down	< 0.001
30328325_Pla	**miR-128-3p**	Up	< 0.05
29966198_Pla	miR-146a-5p	Up	< 0.05
	miR-933	Up	< 0.05
29746584_Ser	miR-103a-3p	Up	< 0.05
	miR-142-3p	Up	< 0.05
	miR-20a-5p	Up	< 0.05
	miR-29b-3p	Up	< 0.05
	let-7b-5p	Up	< 0.05
	let-7 g-5p	Up	< 0.05
	miR-106a-5p	Up	< 0.05
	miR-106b-5p	Up	< 0.05
	miR-18b-5p	Up	< 0.05
	miR-223-3p	Up	< 0.05
	miR-26a-5p	Up	< 0.05
	miR-26b-5p	Up	< 0.05
	miR-301a-3p	Up	< 0.05
	miR-30b-5p	Up	< 0.05
	**miR-132-3p**	Down	< 0.05
	miR-146a-5p	Down	< 0.05
	miR-15a-5p	Down	< 0.05
	**miR-22-3p**	Down	< 0.05
	miR-320a-3p	Down	< 0.05
	miR-320b	Down	< 0.05
	miR-92a-3p	Down	< 0.05
	miR-1246	Down	< 0.05
29635818_Pla	miR-1908-5p	Up	< 0.05
29606187_Ser	miR-135a-5p	Up	< 0.05
	miR-193b-3p	Down	< 0.01
	miR-384	Up	< 0.05
29036829_WB	miR-144-5p	Down	0.03
	miR-374a-5p	Down	0.034
	miR-221-3p	Down	0.042
29036818_Ser_Mild	**miR-106b-3p**	Up	< 0.001
	miR-26a-5p	Down	< 0.001
	**miR-181c-3p**	Down	< 0.001
	**miR-126-5p**	Down	< 0.001
	**miR-22-3p**	Down	< 0.001
	**miR-148b-5p**	Down	< 0.001
29036818_Ser_Mod	**miR-106b-3p**	Up	< 0.001
	miR-1246	Up	< 0.001
	miR-26a-5p	Down	< 0.001
	**miR-181c-3p**	Down	< 0.001
	**miR-126-5p**	Down	< 0.001
	**miR-22-3p**	Down	< 0.001
	**miR-148b-5p**	Down	< 0.001
29036818_Ser_Sev	**miR-106b-3p**	Up	< 0.001
	miR-1246	Up	< 0.001
	miR-660-5p	Up	< 0.001
	miR-26a-5p	Down	0.007
	**miR-181c-3p**	Down	< 0.001
	**miR-126-5p**	Down	< 0.001
	**miR-22-3p**	Down	< 0.001
	**miR-148b-5p**	Down	< 0.001
28934394_Ser	miR-455-3p	Up	0.007
28849039_Ser_Mil	**miR-222-3p**	Down	< 0.05
28849039_Ser_Mod	**miR-222-3p**	Down	< 0.05
28626163_Ser	miR-146a-5p	Up	< 0.05
	**miR-106b-3p**	Up	< 0.05
	miR-195-5p	Up	< 0.05
	miR-20b-5p	Up	< 0.05
	miR-497-5p	Up	< 0.05
	**miR-29c-3p**	Down	< 0.05
	**miR-93-5p**	Down	< 0.05
	miR-19b-3p	Down	< 0.05
	miR-125b-3p	Down	< 0.05
28179587_Pla_AD2	**miR-486-5p**	Up	< 0.001
	**miR-483-5p**	Up	< 0.0001
	**miR-502-3p**	Up	< 0.0001
	**miR-200a-3p**	Up	< 0.01
	miR-151a-5p	Down	< 0.001
	miR-30b-5p	Down	< 0.01
	miR-103a-3p	Down	< 0.01
28179587_Pla_MCI-AD2	**miR-486-5p**	Up	< 0.001
	**miR-483-5p**	Up	< 0.001
	**miR-502-3p**	Up	< 0.01
	**miR-200a-3p**	Up	< 0.05
28137310_Ser	miR-501-3p	Down	0.002
27545218_Ser	miR-613	Up	< 0.01
27501295_WB	miR-9-5p	Down	0.001
	miR-106a-5p	Down	0.001
	miR-106b-5p	Down	0.008
	**miR-107**	Down	0.001
27446280_WB	miR-135b-5p	Down	< 0.01
27277332_WB	miR-206	Up	< 0.001
27239545_PBMC	miR-27b-3p	Up	< 0.05
	**miR-128-3p**	Up	< 0.05
	miR-155-5p	Up	< 0.05
27027823_Ser	miR-125b-5p	Down	< 0.001
	miR-223-3p	Down	< 0.001
	miR-29	Down	< 0.01
	miR-519	Up	< 0.001
26973465_Pla	miR-10b-5p	Down	0.022
	miR-29a-3p	Down	0.041
	miR-130b-3p	Down	0.002
26497032_PBMC	miR-425-5p	Up	< 0.001
	miR-339-5p	Up	0.003
	miR-639	Down	0.04
26078483_Ser	miR-31-5p	Down	< 0.0001
	**miR-93-5p**	Down	< 0.0001
	miR-143-3p	Down	< 0.0001
	miR-146a-5p	Down	< 0.0001
25955795_WB	**miR-29c-3p**	Down	0.0001
25742200_Pla	**miR-107**	Down	< 0.001
25667669_Ser	miR-210-3p	Down	< 0.01
25152461_Ser	miR-135a-5p	Down	< 0.05
	miR-200b-3p	Down	< 0.05
24827165_Pla	miR-384	Down	< 0.05
24827165_Ser	miR-384	Down	< 0.05
24577456_Ser	miR-98-5p	Down	2.67 × 10E−4
	miR-885-5p	Down	2.8 × 10E−4
	miR-483-3p	Down	1.0 × 10E−4
	miR-342-3p	Down	9.19 × 10E−16
	**miR-191-5p**	Down	1.54 × 10E−9
	**let-7d-5p**	Down	1.2 × 10E−6
24550773_Pla	**miR-34c-5p**	Up	< 0.01
24550773_PBMC	**miR-34c-5p**	Up	< 0.01
24157723_Pla	miR-34a-5p	Down	< 0.05
	miR-146a-5p	Down	< 0.05
24139697_Ser	miR-125b-5p	Down	< 0.0001
	miR-181c-5p	Down	< 0.0001
	miR-9-5p	Up	0.0045
24064186_PBMC_Mon	**miR-128-3p**	Up	< 0.05
24064186_PBMC_Lym	**miR-128-3p**	Up	< 0.05
23922807_Pla_C2	**let-7d-5p**	Down	0.0001
	let-7 g-5p	Down	0.001
	miR-15b-5p	Down	0.001
	miR-142-3p	Down	0.0001
	**miR-191-5p**	Down	0.002
	miR-545-3p	Down	0.03
23895045_WB	miR-151a-3p	Up	< 0.05
	let-7d-3p	Up	< 0.05
	miR-5010-3p	Up	< 0.05
	let-7f-5p	Down	< 0.05
	miR-1285-5p	Down	< 0.05
	**miR-107**	Down	< 0.05
	miR-103a-3p	Down	< 0.05
	miR-26b-5p	Down	< 0.05
	miR-26a-5p	Down	< 0.05
	miR-532-5p	Down	< 0.05
23435408_PBMC	miR-29b-3p	Down	0.002
22155483_Ser	miR-137-3p	Down	< 0.05
	miR-181c-5p	Down	< 0.05
	miR-9-5p	Down	< 0.05
	miR-29a-3p	Down	< 0.05
	miR-29b-3p	Down	< 0.05
19936094_PBMC	miR-34a-5p	Up	< 0.05
	miR-181b-5p	Up	< 0.05

**Table 3:** Statistically significant DEmiRNAs identified by the meta-analysis

MiRNAs	Comparison ID	Number of upregulated case in AD	Number of downregulated case in AD	Number of control case	Weight	***P*** value	FDR	LogOR	95% CI	τ^2	***I***^2
miR-107	27501295_WB		172	109	25.08%	3.74E−25	1.61E−23	− 10.40	[− 12.36 , − 8.43]	0.00	0.00%
	25742200_Pla		97	81	25.03%						
	23895045_WB		94	21	24.82%						
	31420923_Pla		120	120	25.07%						
miR-106b-3p	29036818_Ser_Mild	31		86	24.98%	1.05E−20	1.53E−19	9.38	[7.41 , 11.35]	0.00	0.00%
	29036818_Ser_Mod	52		86	25.06%						
	29036818_Ser_Sev	38		86	25.01%						
	28626163_Ser	45		40	24.96%						
miR-22-3p	29036818_Ser_Mild		31	86	24.98%	1.07E−20	1.53E−19	− 9.38	[− 11.34 , − 7.41]	0.00	0.00%
	29036818_Ser_Mod		52	86	25.06%						
	29036818_Ser_Sev		38	86	25.01%						
	29746584_Ser		47	38	24.95%						
miR-126-5p	29036818_Ser_Mild		31	86	33.28%	2.06E−16	1.48E−15	− 9.53	[− 11.81 , − 7.26]	0.00	0.00%
	29036818_Ser_Mod		52	86	33.39%						
	29036818_Ser_Sev		38	86	33.33%						
miR-148b-5p	29036818_Ser_Mild		31	86	33.28%	2.06E−16	1.48E−15	− 9.53	[− 11.81 , − 7.26]	0.00	0.00%
	29036818_Ser_Mod		52	86	33.39%						
	29036818_Ser_Sev		38	86	33.33%						
miR-181c-3p	29036818_Ser_Mild		31	86	33.28%	2.06E−16	1.48E−15	− 9.53	[− 11.81 , − 7.26]	0.00	0.00%
	29036818_Ser_Mod		52	86	33.39%						
	29036818_Ser_Sev		38	86	33.33%						
miR-128-3p	27239545_PBMC	30		30	25.02%	4.64E−16	2.85E−15	8.19	[6.21 , 10.17]	0.00	0.00%
	24064186_PBMC_Mon	34		37	25.08%						
	24064186_PBMC_Lym	34		37	25.08%						
	30328325_Pla	20		20	24.82%						
miR-93-5p	26078483_Ser		79	75	50.13%	2.3E−11	1.24E−10	− 9.50	[− 12.28 , − 6.71]	0.00	0.00%
	28626163_Ser		45	40	49.87%						
miR-29c-3p	25955795_WB		30	30	49.88%	1.81E−09	8.65E−09	− 8.56	[− 11.36 , − 5.77]	0.00	0.00%
	28626163_Ser		45	40	50.12%						
miR-132-3p	29746584_Ser		47	38	50.28%	6.67E−09	2.87E−08	− 8.27	[− 11.07 , − 5.48]	0.00	0.00%
	31849573_Pla		16	31	49.72%						
miR-222-3p	28849039_Ser_Mil		30	30	50.00%	8.09E−09	3.16E−08	− 8.22	[− 11.02 , − 5.43]	0.00	0.00%
	28849039_Ser_Mod		30	30	50.00%						
miR-34c-5p	24550773_Pla	25		27	50.00%	2.67E−08	9.58E−08	7.94	[5.14 , 10.74]	0.00	0.00%
	24550773_PBMC	25		27	50.00%						
let-7d-5p	23922807_Pla_C2		20	17	49.74%	8.9E−06	2.73E−05	− 9.39	[− 13.54 , − 5.25]	4.89	54.62%
	24577456_Ser		158	155	50.26%						
miR-191-5p	23922807_Pla_C2		20	17	49.74%	8.9E−06	2.73E−05	− 9.39	[− 13.54 , − 5.25]	4.89	54.62%
	24577456_Ser		158	155	50.26%						
miR-200a-3p	28179587_Pla_AD2	13		9	50.26%	3.37E−05	8.05E−05	6.01	[3.17 , 8.85]	0.00	0.00%
	28179587_Pla_MCI-AD2	8		9	49.74%						
miR-483-5p	28179587_Pla_AD2	13		9	50.26%	3.37E−05	8.05E−05	6.01	[3.17 , 8.85]	0.00	0.00%
	28179587_Pla_MCI-AD2	8		9	49.74%						
miR-486-5p	28179587_Pla_AD2	13		9	50.26%	3.37E−05	8.05E−05	6.01	[3.17 , 8.85]	0.00	0.00%
	28179587_Pla_MCI-AD2	8		9	49.74%						
miR-502-3p	28179587_Pla_AD2	13		9	50.26%	3.37E−05	8.05E−05	6.01	[3.17 , 8.85]	0.00	0.00%
	28179587_Pla_MCI-AD2	8		9	49.74%						

For subgroup analysis, among 54 comparisons, 7, 17, 23, and 7 comparisons investigated the DEmiRNAs in whole blood, plasma, serum, and PBMC, respectively. The statistically significant DEmiRNAs from the subgroup meta-analysis are shown in Table 4. MiR-107 was consistently found in two subgroups, whole blood and plasma. Two miRNAs, miR-103a-3p in plasma and miR-181c-5p in serum, were found statistically significant in the subgroup analysis, but not in the meta-analysis. MiR-103a-3p is recently reported to be related to AD progression via regulating *NPAS3* expression [96], while low level of miR-181c-5p in serum is suggested to be an indicator for cerebral vulnerability in AD [97].

**Table 4:** Statistically significant DEmiRNAs identified by the meta-analysis in subgroup analysis

Blood elements	miRNA	Comparison ID	Number of upregulated case in AD	Number of downregulated case in AD	Number of control case	Weight	***P*** value	FDR	LogOR	95% CI	τ^2	***I***^2
Whole blood	miR-107	27501295_WB		172	109	50.26%	1.03E−12	2.06E−12	− 10.12	[− 12.91, − 7.34]	0.00	0.00%
		23895045_WB		94	21	49.74%						
Plasma	miR-103a-3p	28179587_Pla_AD2		13	9	49.64%	0.000266	0.00031	− 8.62	[− 13.26, − 3.99]	7.09	63.36%
		31420923_Pla		120	120	50.36%						
	miR-107	25742200_Pla		97	81	49.96%	5.26E−14	3.68E−13	− 10.67	[− 13.45, − 7.89]	0.00	0.00%
		31420923_Pla		120	120	50.04%						
	miR-200a-3p	28179587_Pla_AD2	13		9	50.26%	3.37E−05	4.72E−05	6.01	[3.17, 8.85]	0.00	0.00%
		28179587_Pla_MCI-AD2	8		9	49.74%						
	miR-483-5p	28179587_Pla_AD2	13		9	50.26%	3.37E−05	4.72E−05	6.01	[3.17, 8.85]	0.00	0.00%
		28179587_Pla_MCI-AD2	8		9	49.74%						
	miR-486-5p	28179587_Pla_AD2	13		9	50.26%	3.37E−05	4.72E−05	6.01	[3.17, 8.85]	0.00	0.00%
		28179587_Pla_MCI-AD2	8		9	49.74%						
	miR-502-3p	28179587_Pla_AD2	13		9	50.26%	3.37E−05	4.72E−05	6.01	[3.17, 8.85]	0.00	0.00%
		28179587_Pla_MCI-AD2	8		9	49.74%						
Serum	miR-106b-3p	29036818_Ser_Mild	31		86	24.98%	1.05E−20	9.08E−20	9.38	[7.41, 11.35]	0.00	0.00%
		29036818_Ser_Mod	52		86	25.06%						
		29036818_Ser_Sev	38		86	25.01%						
		28626163_Ser	45		40	24.96%						
	miR-126-5p	29036818_Ser_Mild		31	86	33.28%	2.06E−16	7.01E−16	− 9.53	[− 11.81, − 7.26]	0.00	0.00%
		29036818_Ser_Mod		52	86	33.39%						
		29036818_Ser_Sev		38	86	33.33%						
	miR-148b-5p	29036818_Ser_Mild		31	86	33.28%	2.06E−16	7.01E−16	− 9.53	[− 11.81, − 7.26]	0.00	0.00%
		29036818_Ser_Mod		52	86	33.39%						
		29036818_Ser_Sev		38	86	33.33%						
	miR-181c-3p	29036818_Ser_Mild		31	86	33.28%	2.06E−16	7.01E−16	− 9.53	[− 11.81, − 7.26]	0.00	0.00%
		29036818_Ser_Mod		52	86	33.39%						
		29036818_Ser_Sev		38	86	33.33%						
	miR-181c-5p	24139697_Ser		105	155	50.39%	0.00354	0.008598	− 8.28	[− 13.84, − 2.71]	11.96	74.29%
		22155483_Ser		7	7	49.61%						
	miR-22-3p	29036818_Ser_Mild		31	86	24.98%	1.07E−20	9.08E−20	− 9.38	[− 11.34, − 7.41]	0.00	0.00%
		29036818_Ser_Mod		52	86	25.06%						
		29036818_Ser_Sev		38	86	25.01%						
		29746584_Ser		47	38	24.95%						
	miR-93-5p	26078483_Ser		79	75	50.13%	2.30E−11	6.53E−11	− 9.50	[− 12.28, − 6.71]	0.00	0.00%
		28626163_Ser		45	40	49.87%						
	miR-222-3p	28849039_Ser_Mil		30	30	50.00%	8.09E−09	2.08E−08	− 8.22	[− 11.02, − 5.43]	0.00	0.00%
		28849039_Ser_Mod		30	30	50.00%						
PBMC	miR-128-3p	27239545_PBMC	30		30	33.28%	3.94E−13	3.94E−13	8.44	[6.16, 10.72]	0.00	0.00%
		24064186_PBMC_Mon	34		37	33.36%						
		24064186_PBMC_Lym	34		37	33.36%						

### Identification of potential biomarkers by the ABMDA ensemble learning method

A total of 1751 known miRNA–disease associations between 413 miRNAs and 227 diseases were obtained from the “circulation_biomarker_diagnosis” category in HMDD, including 17 known miRNA–AD associations. Identification of miRNA–disease associations by meta-analysis increased the number of positive miRNA–AD associations by 15 for the ABMDA identification after removing duplicates. The eleventh miRNA from the ABMDA results was determined to be statistically insignificant in the meta-analysis. Therefore, the first 10 miRNAs with prediction scores ranging from 9.45 to 7.88 were collected (Table 5; Additional file 2). Most of these 10 miRNAs have been associated with multiple types of cancer as diagnostic or prognostic biomarkers [98–104]. Only one miRNA, miR-155, has been reported as a biomarker in AD for mediating neuroinflammation [105].

**Table 5:** AD-related DEmiRNAs identified by ABMDA

Disease	miRNAs	Mature miRNAs	Score	Used as biomarkers in diseases
Alzheimer disease	miR-339	miR-339-5p	9.45	Lung cancer
	miR-128-2		8.92	Hepatocellular carcinoma
	miR-203	miR-203a-3p	8.76	
	miR-495	miR-495-3p	8.75	Non-small cell lung cancer
	miR-155	miR-155-5p	8.70	AD
	let-7a-2		8.67	Lung cancer
	miR-103a-2		8.10	
	miR-16-2		8.02	Breast cancer
	let-7b	let-7b-5p	7.93	Non-small cell lung cancer
	miR-625	miR-625-5p	7.88	Malignant pleural mesothelioma

MiR-339-5p is upregulated to alleviate neuroinflammation by inhibiting *HMGB1* [106]. *HMGB1* encodes high mobility group box 1 to produce proinflammatory cytokines by binding to receptors for advanced glycation end products (RAGE) [107] and inhibiting IKK-β and IKK-γ, which are key elements of NF-κB signaling [108]. NF-κB signaling can also be modulated by the identified DEmiRNAs through multiple mechanisms. Both miR-203a-3p and let-7b-5p target *IGF1R*, which encodes insulin-like growth factor 1 receptor, to alleviate tumor necrosis factor (TNF)-induced activation of NF-κB [109, 110]. MiR-155 and miR-625-5p decrease the expression of *SHIP* and *AKT2*, which encode Src homology 2-containing inositol phosphatase and RAC-beta serine/threonine-protein kinase, respectively, to attenuate NF-κB-dependent inflammation [111, 112]. Additionally, miR-495 targets *NOD1*, which encodes nucleotide-binding oligomerization domain-containing protein 1, to reduce high glucose-induced inflammation in diabetic complications [113], whereas its mature form, miR-495-3p, has recently been reported to regulate inflammatory molecules by targeting *IL5RA* [114]. The mature forms of miR-128-2, let-7a-2, miR-103a-2, and miR-16-2 have not been reported in the miRBase database at the time of this study; however, their family members exhibit inflammatory properties. MiR-128 and let-7a regulate gene expression in response to oxidative stress [115, 116]. MiR-16 and miR-103-3p target *ADORA2A* and *SNRK*, which encode the adenosine A2a receptor and sucrose non-fermentable-related serine/threonine-protein kinase, respectively, to attenuate NF-κB-dependent inflammation [117, 118]. Current literature suggests that NF-κB might be the main downstream effector for the DEmiRNAs identified by ABMDA in the present study.

### Enrichment analysis

**Fig. 3:**
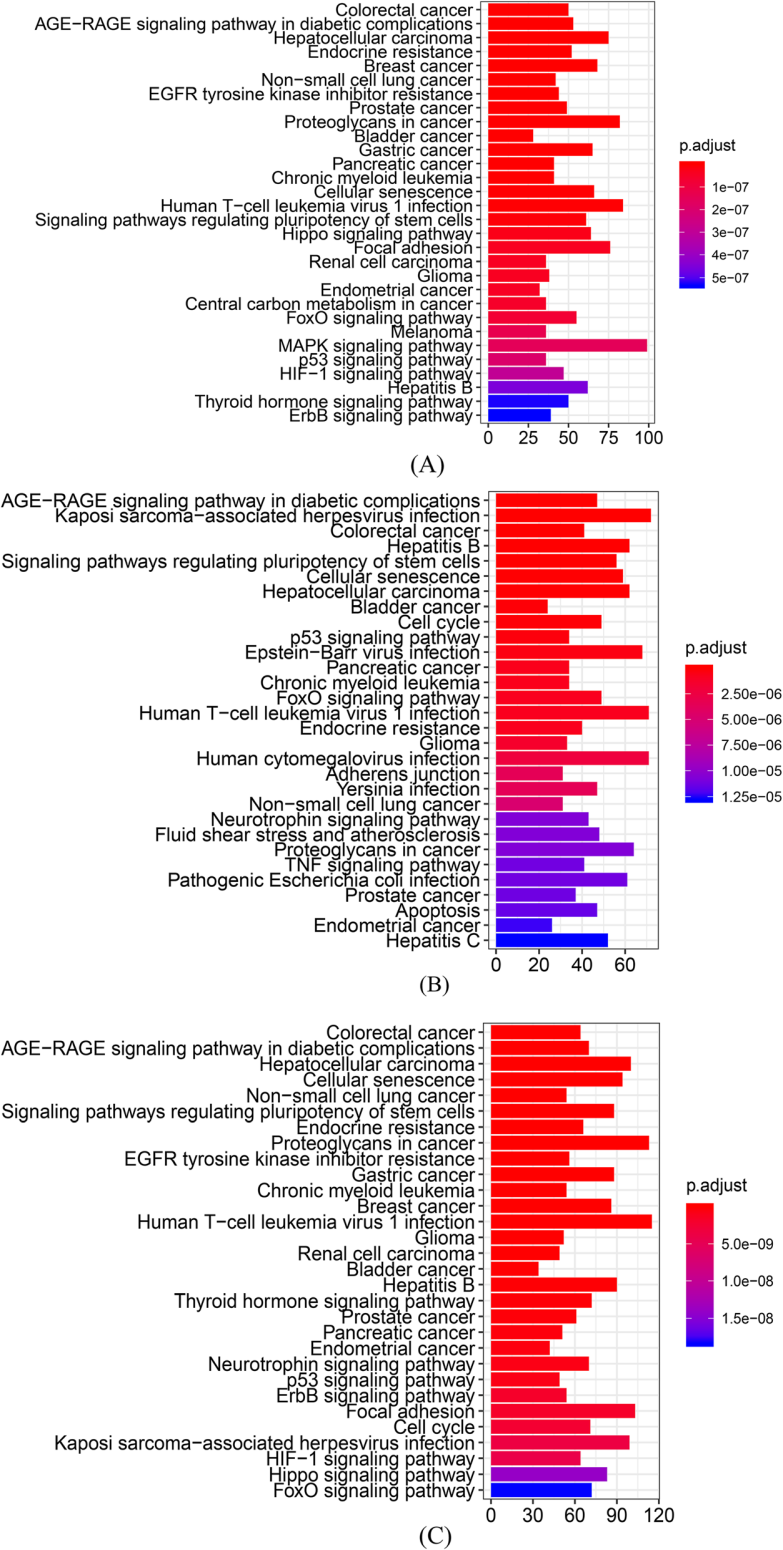
Biological pathway enrichment analysis of the target genes of DEmiRNAs identified by **A** meta-analysis; **B** ABMDA; and **C** both the meta-analysis and ABMDA. The x-axis represents the number of genes in each of the KEGG pathway, the y-axis represents the name of each KEGG pathway, and the color represents the FDR-adjusted statistical significance

There were 3496 and 2938 target genes in total for 18 and 10 DEmiRNAs from the meta-analysis and ABMDA results, respectively (Additional file 3). These target genes were subjected to two independent enrichment analyses based on KEGG to obtain the functional annotations of the DEmiRNAs (Fig. 3A, B). Most pathways targeted by the meta-analysis and ABMDA DEmiRNAs were commonly found in the two independent enrichment analyses, even though the DEmiRNAs in the two categories were not identical. This indicated that multiple DEmiRNAs target either common or different mRNA transcripts that functionally converge on the same pathways. Most dysregulated pathways in the enrichment analysis were involved in AD development via modulation of neuroinflammation, including the AGE-RAGE signaling pathway in diabetic complications, cell cycle, cellular senescence, Hippo signaling pathway, and FoxO signaling pathways. In addition, multiple cancerous pathways were also implicated. Compared with the two independent enrichment analyses, the synergic enrichment analysis unifying the two categories of DEmiRNAs might provide better insight for AD development. In the synergic enrichment analysis, the dysregulated pathways were more statistically significant (Fig. 3C). Also, the pathways that were found in only one independent enrichment analysis (such as the Hippo signaling pathway from the meta-analysis and cell cycle from ABMDA) were both statistically significant in the synergic enrichment analysis. The synergic enrichment analysis suggested that the two DEmiRNA categories interact functionally and complement each other.

In the synergic enrichment analysis, the AGE-RAGE signaling pathway in diabetic complications was identified as the second most statistically significant. The AGE-RAGE pathway is involved in diabetic microvascular complications. Elevated levels of AGE and RAGE have also been reported in AD patients, and increased RAGE activity has been detected in patients with early AD symptoms [119, 120]. RAGE also interacts with Aβ oligomers to induce BBB leakage [121] and upregulates NF-κB, which induces neuroinflammation [122]. Elevated neuroinflammation increases the expression of secretases for Aβ production [123], reduces Aβ degradation in microglia [124], and induces the aberrant hyperphosphorylation of tau proteins [125]. Elevated levels of proinflammatory cytokines and Aβ increase cell cycle-related kinases, such as PKA, CAMKII, Fyn, and mTORC1, inducing neuronal cell cycle re-entry (CCR) [126, 127]. Aberrant CCR results in neuronal hyperploidy, which alters neuronal circuit function and reduces synaptic activity [128, 129], ultimately inducing neuronal death [130]. Aberrant CCR is also induced by malfunction of PI3K/AKT/mTOR, a cell survival pathway disrupted in both AD and cancer, though in opposite directions [131]. Further, aberrant CCR is a causative factor for the majority of neuronal death in early AD development and might be a potential biological mechanism to link AD and multiple cancerous diseases. Aberrant Aβ accumulation and proinflammatory cytokines from dying neurons further enhance neuroinflammation and oxidative stress, inducing cellular senescence and dysregulating the Hippo and FoxO signaling pathways. Cellular senescence is a permanent state of cellular rest that is involved in the onset of AD [132], and neuroinflammation and Aβ-mediated toxicity have been reported to upregulate senescence-regulated genes [133–135]. The Hippo signaling pathway is a kinase cascade relevant for cellular homeostasis, and is upregulated by Aβ-mediated neurotoxicity to enhance neurodegeneration with JNK in AD [136, 137]. The FoxO signaling pathway is involved in the relationship between ROS, insulin resistance, and AD pathology [138, 139]. Under persistent oxidative stress, the FoxO signaling pathway increases the transcription of apoptotic proteins [140].

### Network

In total, 5222 target genes were identified for the DEmiRNAs from the meta-analysis and ABMDA, and 1865 target genes with semantic similarity over 0.95 were identified as common target genes in the two DEmiRNA categories, suggesting an overlap of the two categories in biological functions. The common target genes were used to retrieve the corresponding protein–protein interactions according to STRING and construct a network. The network comprised 1865 common target genes as nodes, and 18750 edges among the common target genes. The DEmiRNAs let-7b-5p and miR-155 identified by ABMDA shared the most common target genes with the DEmiRNAs miR-93-5p and miR-128-3p identified by the meta-analysis. The common target genes *UBC*, *UBB*, and *RPS27A*, which are core members in the ubiquitin-proteasome system (UPS), exhibited the highest connection degree in the network.

The UPS is imperative not just in Aβ clearance [141], but also in neuroinflammation and neuronal CCR. The physiological function of the UPS can be adversely influenced by neuroinflammation. Under neuroinflammation, J2 prostaglandins are generated from prostaglandin D2, which is the most abundant prostaglandin in the brain [142]. J2 prostaglandins enhance the expression levels of *COX2* to transition acute neuroinflammation to chronic neuroinflammation and oxidize the UPS units to promote disassembly [143]. The impaired UPS induces ectopic expression of cell cycle-related genes and causes neuronal CCR, as the metabolisms of cyclin and cyclin-dependent kinases are dependent on the UPS. A recent study [144] has reported that the dysregulation of an E3 ubiquitin ligase, Itch, induces neuronal CCR in response to Aβ. Aβ-induced JNK activation phosphorylates Itch to promote the degradation of TAp73, which is important for protein synthesis under oxidative stress, in neurons [145].

### Quality assessment of studies

**Fig. 4:**
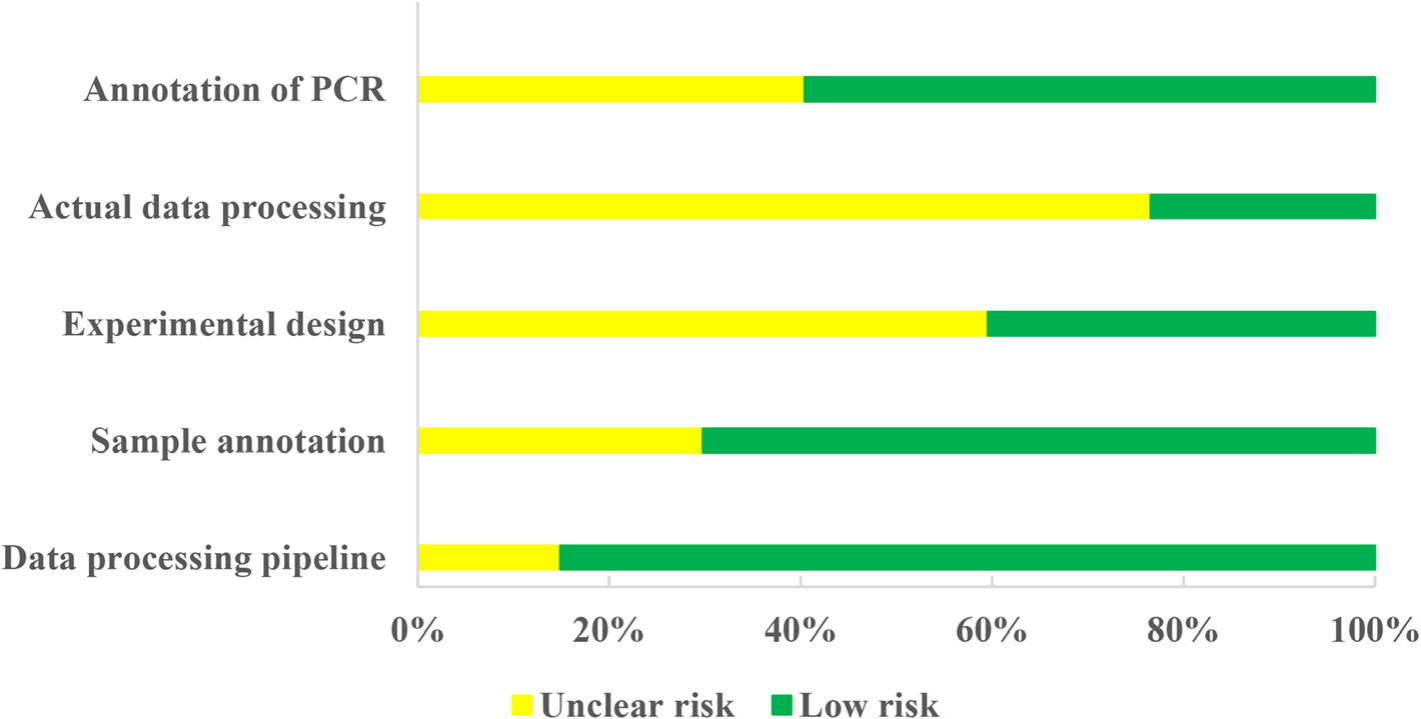
Overall quality assessment of the miRNA differential expression profiling approach for 47 included studies. Green color represents a low risk of bias, in which the authors clearly provided full details of the methods. Yellow color represents an unclear risk of bias, in which the authors provided methods without full details

The MIQE guideline was used to access the expression profiling analysis of the included studies. The results of the quality assessments were shown in Fig. 4. Among the 47 expression profiling studies, miR-39 and U6 RNAs were frequently used as internal normalization controls for qRT-PCR. Around 85% of the included studies provided sufficient information about data processing, including statistical analysis and quantification methods. About 70% of the included studies provided sufficient information about sample annotation, but 16 studies did not provide details of the storage or extraction methods of serum or plasma. Approximately 60% of the included studies did not provide the number of replicates in the experimental design, and 77% did not provide the quantification cycle value in the actual data processing. The parameters of qRT-PCR methods were missing in 14 studies. For the annotation of PCR, most studies provided the full details of reference miRNAs for quantification, but primer information was missing in 19 studies.

## Discussion

There is a need for blood circulating biomarkers that can be mass screened accurately and conveniently to identify high risk individuals of AD. The identification of DEmiRNAs as biomarkers from differential miRNA expression studies has been successful in cancers [146] and is thought to have potential for AD. DEmiRNAs associated with AD pathology, such as Aβ production and neuroinflammation, are potentially important biomarkers because the presence of Aβ and proinflammatory cytokines are considered to be key factors for predicting whether patients with mild cognitive impairment are progressing to AD [147–149]. In this study, we used a meta-analysis approach based on differential miRNA expression studies from blood to identify reliable miRNA–AD associations. The associations were subsequently used for the prediction of potential AD biomarkers using the ABMDA ensemble learning method. We identified 28 DEmiRNAs (18 and 10 from meta-analysis and ABMDA, respectively) as potential AD biomarkers in blood.

The DEmiRNAs identified with the meta-analysis involved in Aβ metabolism, including *APP* expression, Aβ-production enzyme regulation, and Aβ clearance, tau protein phosphorylation, and also contribute to neuronal function during AD progression, including pathogenic neuroinflammation, apoptosis, mitochondrial oxygen chain activity, and neuronal microtubule maintenance. The meta-analytical results of DEmiRNAs mediate Aβ synthesis via several targets, and some of them are also involved in tau protein phosphorylation and neuronal functions. MiR-107 is negatively correlated with *BACE1* and *ADAM10* expression and is downregulated in the early stage of AD [150, 151]. The downregulation of miR-107 also dysregulates the expression of *CDK5R1*, which is involved in neuronal survival [94]. Downregulation of miR-181c dysregulates the expression of *SPTLC1* [152], to increase Aβ deposition, and increases pro-inflammatory cytokines [153]. MiR-22-3p and miR-29c also regulate Aβ deposition via targeting *MAPK14* and *BACE1*, respectively [154, 155]. Besides Aβ synthesis, Aβ clearance is also interfered by the meta-analytical results of DEmiRNAs. MiR-128 and miR-93 are reported to be involved in Aβ phagocytosis and UPS for Aβ clearance by targeting cathepsin and *NEDD4L*, respectively [87, 156]. For Aβ-induced toxicity, upregulation of miR-34c enhances Aβ-induced synaptic failure to suppress the memory formation by targeting *SIRT1* and *VAMP2* [157, 158]. MiR-200a-3p coregulates *BACE1* and *PRKACB* to protect neurons against Aβ-induced toxicity and tau protein hyperphosphorylation [159], while upregulation of miR-200a-3p also interferes the function of mitochondrial oxidative chain [69]. The meta-analytical results of DEmiRNAs were also involved in the tau protein phosphorylation. MiR-132 is consistently downregulated in different brain area, and negatively correlated with Braak stage, suggesting that it has an important role in cognitive capacity and correlated with tau protein phosphorylation [160, 161]. The downregulation of miR-132 is reported to increase the expression of *ITPKB* and *SIRT1* for tau pathology and Aβ generation, respectively [162, 163]. Meanwhile, miR-132 is also involved in caspase 3-dependent apoptosis [164]. MiR-483-5p and miR-106b target *MAPT* and *FYN* for tau protein synthesis and phosphorylation, respectively [69, 165]. For neuronal function, miR-222 targets *CDKN1B* to influence cell cycle and apoptosis [68]; miR-191 targets *TMOD2* and *REST* to regulate axonal guidance and dendritic growth [166]; and both miR-486-5p and miR-502-3p are the regulators of dynactin for neuronal function [69]. The potential mechanisms of miR-126-5p, miR-148-5p, and let-7d in AD are not well known. MiR-126-5p targets JNK-interacting protein 2 to mediate inflammatory response in infectious conditions [167]. The expression of miR-148-5p is reported to be positively correlated with MMSE score [66], and let-7d is reported to be involved in neuronal cell cycle by regulating enzymatic signaling [168]. Compared with other let-7 family members, let-7d does not significantly trigger the release of TNF-α from microglia [169].

DEmiRNAs from ABMDA also relate to neuroinflammation mainly via modulation of NF-κB. The DEmiRNAs identified in the present study are compatible with the first two differential miRNA expression studies in AD, which reported that the DEmiRNAs are associated with dysregulated inflammation [23, 170]. Independent enrichment analysis in the present study indicated disrupted signaling of AGE-RAGE, cellular senescence, Hippo, FoxO, and cell cycles, and these dysregulated pathways were more statistically significant in the synergic enrichment analysis. The dysregulated pathways were associated with neuroinflammation, in which neuroinflammation increases the production of Aβ by modulation of secretase, suggesting a biological significance of the 28 DEmiRNAs in AD development. The similarity of biological functions of the two DEmiRNA categories fits the ABMDA assumption that the miRNAs associated with the same disease should be functionally related. In the network, 1865 target genes were commonly found from the two DEmiRNA categories, and the highly connected target genes involved *UBC*, *UBB*, and *RPS27A*, which are involved in the neuroinflammatory process and CCR through mediating the UPS.

**Fig. 5:**
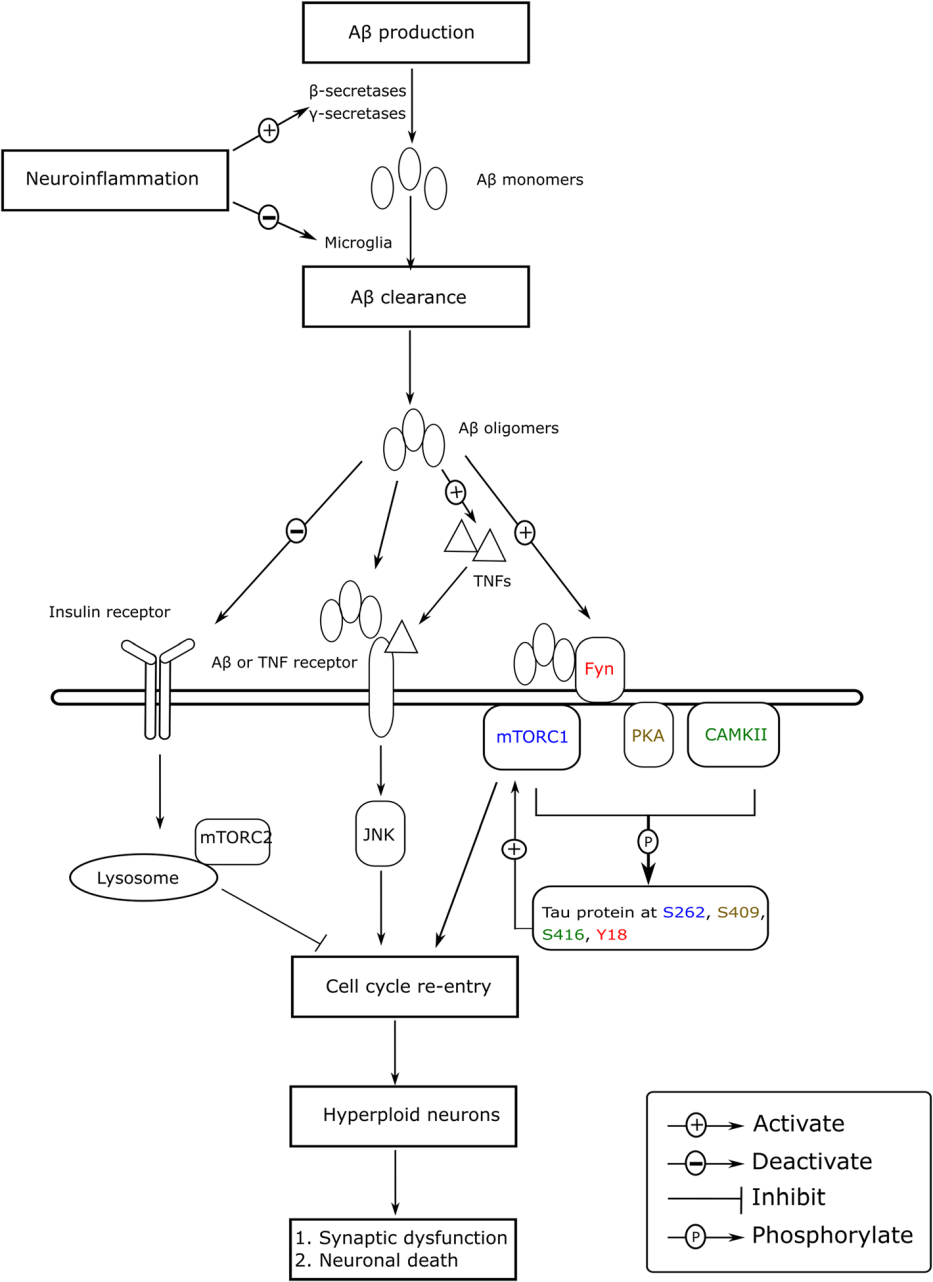
Mechanism of action of neuronal CCR in AD

Comparison of DEmiRNA results from the meta-analysis and ABMDA revealed that the cell cycle pathway was identified as significant only with ABMDA. The majority of neuronal death in AD is due to the dysregulated cell cycle, in which the differentiated neurons in AD-affected brain regions re-enter the cell cycle in the presymptomatic disease [171, 172]. The ectopic expression of developmentally regulated genes in AD links AD pathophysiology with aberrant CCR and correlates with cognitive decline [173]. CCR markers are expressed in Aβ-cultured neurons within hours, suggesting CCR is an initial event in response to Aβ [174]. These findings indicate that neuronal CCR induces rapid neuronal loss after exposure to Aβ. Neuronal CCR in AD results from Aβ-induced activation of multiple protein kinases at the plasma membrane, and tau protein phosphorylation by these proteins. The absence of β-secretases, or blockage of the Aβ receptor, inhibits CCR [175, 176]. Aβ incorporates into the lipid rafts of neuronal membranes by Fyn-dependent kinase [177] and disturbs the structure of lipid rafts to activate PKA, CAMKII, Fyn, and mTORC1 to phosphorylate tau proteins at S409, S416, Y18, and S262, respectively [126, 127]. The phosphorylated tau proteins subsequently modify mTORC1 activity to induce CCR. Aβ also induces a rapid loss of the insulin receptor in the brain and impairs insulin receptor autophosphorylation to reduce brain insulin signaling [178], decreasing the inhibitory effects of mTORC1 at lysosomes in Aβ-induced CCR [179, 180]. CCR can also be initiated by JNK, which is activated when Aβ binds to receptors, such as RAGE, or by Aβ-induced TNF [144, 181]. Once CCR is initiated, neurons do not complete the cell cycle [171, 174]. The hyperploidy of neurons results in hypertrophy of neuronal cell bodies in AD-affected regions [182, 183]. The number of hyperploid neurons is higher in preclinical AD individuals than in healthy individuals, and initially increases followed by a gradual decrease in AD development [171]. The hyperploidy gradually decreases the synaptic inputs, which correlates with reduced activity of PSD-95 [129], a scaffold protein for glutamatergic function, suggesting that CCR-induced hyperploidy results in synaptic dysfunction. Further, loss of chromosomal homeostasis induces neuronal death [184], probably via neurotrophins and activation of FOXO1. Neurotrophins, such as nerve growth factor (NGF) and brain-derived neurotrophic factor (BDNF), elicit prosurvival functions, whereas their precursors, proNGF and proBDNF, elicit apoptotic functions. The levels of neurotrophins and their precursors are downregulated and upregulated in AD, respectively [185, 186], shifting neurotrophic prosurvival functions to apoptotic functions. Additionally, CCR induces dysregulation of cyclin metabolism to activate the transcription factor, FOXO1. The cyclin B-CDK1 complex, which is upregulated in AD, phosphorylates FOXO1 [174] to induce the expression of apoptotic genes [187]. Thus, CCR is a key mechanism to functionally connect Aβ and tau proteins in the early phase of AD development and connects the seemingly unrelated pathologies between AD and cancer, and between AD and insulin signaling impairment [188]. This convergence of pathology points to the importance of CCR in AD development and significance of miRNAs involved in CCR. Figure 5 summarizes the mechanism of Aβ-mediated CCR in AD, as described above.

Although it remains unclear whether neuroinflammation is the primary cause of AD development or a secondary event to other primary pathologies, neuroinflammation is an imperative and early event for Aβ-mediated toxicity and has been suggested to contribute as much as Aβ in AD pathology [189]. The level of neuroinflammation assessed by microglial activation has been correlated with worse cognitive decline in a PEG imaging study of AD participants [190]. Neuroinflammation is progressively increased during AD development, as indicated by elevated levels of proinflammatory cytokines [191]. Proinflammatory cytokines have been identified around amyloid plaques [192], suggesting a role of neuroinflammation in Aβ deposition. Neuroinflammation impairs the microglial Aβ degradation ability [193]. Several genome-wide association studies have reported that immune-related genes are involved in AD pathogenesis [194–197], such as *CD33*, *CR1*, *EPHA1*, and *MS4A6E/MS4A4E*, which regulate the immune system in response to Aβ and activate microglial Aβ degradation. An epigenome-wide association study [198] demonstrated hypermethylation of *ANK1* in both presymptomatic and AD patients. *ANK1* encodes Ankyrin 1 to maintain the actin cytoskeleton, and the upregulation of ANK1 in AD microglia has been reported, which supports the significance of the microglial response in AD development [199]. Passamonti et al. [200] reported that cognitive decline is mediated by microglial activation, which is linked to neuroinflammation. The perpetuation of inflammation influences synaptic plasticity and induces neuronal damage, resulting in neurodegeneration [201]. Synaptic loss results primarily from the increased levels of proinflammatory cytokines and activated microglial cells [202]. However, the importance of neuroinflammation in AD has been questioned because of the failure of nonsteroidal anti-inflammatory drugs (NSAIDs) for treating AD. An early AD clinical trial reported the failure of both cyclooxygenase-nonselective (naproxen) and cyclooxygenase-selective (celecoxib) NSAIDs in cognitive function improvement [203]. Another study [204], which involved long-term follow-up of AD participants who received naproxen or celecoxib, reported that naproxen reduces AD incidence in asymptomatic individuals. The trials suggest that the efficacy of NSAIDs is disease stage dependent and that the choice of NSAID is a determining factor. A COX nonselective NSAID, tarenflurbil, has shown some positive results at a high dosage for individuals with mild symptoms in a post hoc analysis of a phase II clinical trial [205], but no cognitive improvement was observed with the drug in a phase III clinical trial [206]. One possible reason for its failure is the low penetration of the drug from plasma to CSF. A recent study indicated that intranasal delivery of tarenflurbil can increase the drug concentration in the brain [207], but no clinical trials have yet been carried out with this route of delivery.

The miRNA expression profile in blood is representative of dysregulation in all tissues. The DEmiRNAs could be biased by the presence of multiple comorbidities, although the extent of how specific comorbidities influence DEmiRNAs in AD is not fully known. The miRNAs encapsulated in membrane vesicles can penetrate the BBB [208], suggesting that DEmiRNAs induced by peripheral complications could also contribute to AD pathology in the brain. Peripheral inflammation interferes with immunological processes in the brain through the entry of activated peripheral immune cells that exacerbate or initiate neuroinflammation. A growing body of evidence supports that peripheral inflammation is a driver of AD. For example, obesity and type 2 diabetes mellitus increase the risk of AD development through mediating neuroinflammation [209], and the gut microbial diversity in obesity patients is associated with increased levels of proinflammatory cytokines in blood [210]. The AD pathogenetic factor *APOE* ε4 allele has been reported to impair macrophage efferocytosis, which subsequently induces tissue inflammation and increases the circulating levels of proinflammatory cytokines [211, 212]. Elevated levels of proinflammatory cytokines in the blood of AD patients have been reported in meta-analyses [213, 214], confirming the presence of peripheral inflammation in AD. Peripheral inflammation is positively associated with cognitive decline and could detrimentally impact brain function [215]; specifically, elevated systemic TNF levels have been associated with increased conversion of mild cognitive impairment to AD. Systemic TNFs bind to TNF receptors to alter BBB integrity and allow entry of peripheral immune cells to the brain, inducing neuroinflammation [216, 217]. The entry of peripheral immune cells disrupts synaptic plasticity to induce neuroinflammation, increasing the risk of cognitive decline [215, 218]. Neuroinflammation also releases antigens to activate T cells in CSF. The activated T cells enter the brain and differentiate into effector T cells, which can produce cytokines [219] or induce apoptosis [220]. Abnormalities in T cells have been reported in AD patients compared with controls [221].

### Limitations

Current approaches to diagnose AD (e.g., FDG-PET and CSF-based Aβ and tau protein levels) require sophisticated equipment or lumbar puncture, which could be avoided with the use of blood-based biomarkers. The strength of this study is the integration of meta-analysis and the ABMDA ensemble learning method to identify potential AD biomarkers in blood. However, there are some limitations. First, most of the included miRNA expression studies only conducted qRT-PCR as a validation approach to identify DEmiRNAs, and only 17 of the included studies reported both screening and validation approaches. This means that the present identification of DEmiRNAs was dependent on those selected by researchers in previous studies, and not an objective manner. This implies that the number of DEmiRNAs in AD is likely larger than the number of DEmiRNAs identified in the present study. Second, although most DEmiRNAs from the meta-analysis and ABMDA identification were associated with neuroinflammation, their function was not distinguishable between acute and chronic neuroinflammation. Acute neuroinflammation is beneficial for microglial clearance of Aβ, whereas chronic neuroinflammation results in neurodegeneration by mediating functional and structural damage to neurons [222, 223]. Third, there were 9 DEmiRNAs from the meta-analysis extracted from the independent comparisons of only one study, suggesting that the meta-analytical results might be biased by the unidentified correlation within the recruited population. Forth, disease stages of AD patients were not fully and consistently provided in most included differential miRNA expression studies, resulting in the performance of subgroup analysis based on disease stage was not currently applicable. Also, the results of original studies might be influenced by the lack of adjustment for the disease stages. Fifth, all the included differential miRNA expression studies were non-longitudinal studies, suggesting that the miRNAs extracted from these studies were not repeated observations over periods of time.

## Conclusion

This study identified 28 DEmiRNAs as potential AD biomarkers in blood by meta-analysis and ABMDA ensemble learning in tandem. The DEmiRNAs identified by both meta-analysis and ABMDA were related to neuroinflammation, and those identified solely by ABMDA were related to neuronal CCR.

## Supplementary Information

**Additional file 1.** The meta-analysis results of DEmiRNAs.

**Additional file 2.** The ABMDA results.

**Additional file 3.** The target genes of the DEmiRNAs identified by meta-analysis and ABMDA.

## Data Availability

The raw data of this paper are available in the public database, PubMed. The data that support the findings of this paper are available in the published article and its additional files.

## Abbreviations

Aβ: Amyloid-beta
ABMDA: Adaptive boosting for miRNA disease association
AD: Alzheimer’s disease
AGE: Advanced glycation end product
BBB: Blood-brain barrier
BDNF: Brain-derived neurotrophins
CCR: Cell cycle re-entry
CI: Confidence interval
CSF: Cerebrospinal fluid
DEmiRNAs: Differentially expressed microRNAs
FDG-PET: Fluorodeoxyglucose-positron emission tomography
FDR: False discovery rate
KEGG: Kyoto Encyclopedia of Genes and Genomes
logORs: Log odds ratios
miRNAs: MicroRNAs
MIQE: Minimum Information for Publication of Quantitative Real-time PCR Experiments
MMSE: Mini-Mental State Examination
NGF: Nerve growth factor
NIA-AA: National Institute of Aging and Alzheimer’s Association
NINCDS-ADRDA: National Institute of Neurological and Communicative Disorder and Stroke and the Alzheimer’s Disease and Related Disorders
NSAIDs: Nonsteroidal anti-inflammatory drugs
PBMC: Peripheral blood mononuclear cell
RAGE: Receptor for AGE
qRT-PCR: Quantitative real-time polymerase chain reaction
TNFs: Tumor necrosis factors
UPS: Ubiquitin-proteasome system
τ^2^: Tau square

## References

[1] Guo T, Zhang D, Zeng Y, Huang TY, Xu H, Zhao Y (2020). Molecular and cellular mechanisms underlying the pathogenesis of Alzheimer’s disease. Mol Neurodegener.

[2] Zhao Y, Lukiw WJ (2018). Bacteroidetes neurotoxins and inflammatory neurodegeneration. Mol Neurobiol.

[3] Andrade-Moraes CH, Oliveira-Pinto AV, Castro-Fonseca E, daSilva CG, Guimaraes DM, Szczupak D (2013). Cell number changes in Alzheimer’s disease relate to dementia, not to plaques and tangles. Brain..

[4] Thambisetty M, Lovestone S (2010). Blood-based biomarkers of Alzheimer’s disease: challenging but feasible. Biomark Med.

[5] Dubois B, Feldman HH, Jacova C, deKosky ST, Barberger-Gateau P, Cummings J (2007). Research criteria for the diagnosis of Alzheimer’s disease: revising the NINCDS–ADRDA criteria. Lancet Neurol.

[6] Jack CR, Bennett DA, Blennow K, Carrillo MC, Dunn B, Haeberlein SB (2018). NIA-AA Research Framework: toward a biological definition of Alzheimer’s disease. Alzheimers Dement.

[7] Agrawal M, Biswas A. Molecular diagnostics of neurodegenerative disorders. Front Mol Biosci. 2015;2. Available from: https://www.frontiersin.org/articles/10.3389/fmolb.2015.00054/full. Accessed 22 Sept 2015. 10.3389/fmolb.2015.00054 PMC4585189 26442283

[8] McKhann GM, Knopman DS, Chertkow H, Hyman BT, Jack CR, Kawas CH (2011). The diagnosis of dementia due to Alzheimer’s disease: recommendations from the National Institute on Aging-Alzheimer’s Association workgroups on diagnostic guidelines for Alzheimer’s disease. Alzheimers Dement.

[9] Schindler SE, Fagan AM. Autosomal dominant Alzheimer disease: a unique resource to study CSF biomarker changes in preclinical AD. Front Neurol. 2015;6. Available from: https://www.frontiersin.org/articles/10.3389/fneur.2015.00142/full. Accessed 29 June 2015. 10.3389/fneur.2015.00142 PMC4483518 26175713

[10] Ritchie C, Smailagic N, Noel-Storr AH, Ukoumunne O, Ladds EC, Martin S (2017). CSF tau and the CSF tau/ABeta ratio for the diagnosis of Alzheimer’s disease dementia and other dementias in people with mild cognitive impairment (MCI). Cochrane Database Syst Rev.

[11] Fei M, Jianghua W (2011). RujuanM, Wei Z, QianW. The relationship of plasma Aβ levels to dementia in aging individuals with mild cognitive impairment. J Neurol Sci.

[12] Koyama A, Okereke OI, Yang T, Blacker D, Selkoe DJ, Grodstein F (2012). Plasma amyloid-β as a predictor of dementia and cognitive decline: a systematic review and meta-analysis. Arch Neurol.

[13] Mattsson-Carlgren N, Janelidze S, Palmqvist S, Cullen N, Svenningsson AL, Strandberg O (2020). Longitudinal plasma p-tau217 is increased in early stages of Alzheimer’s disease. Brain.

[14] Shin WS, Di J, Murray KA, Sun C, Li B, Bitan G, et al. Different amyloid-β self-assemblies have distinct effects on intracellular tau aggregation. Front Mol Neurosci. 2019;12. Available from: https://www.frontiersin.org/article/10.3389/fnmol.2019.00268/full. Accessed 8 Nov 2019. 10.3389/fnmol.2019.00268 PMC6856013 31787880

[15] Patel S, Shah RJ, Coleman P, Sabbagh M (2011). Potential peripheral biomarkers for the diagnosis of Alzheimer’s disease. Int J Alzheimers Dis.

[16] O’Brien J, Hayder H, Zayed Y, Peng C. Overview of microRNA biogenesis, mechanisms of actions, and circulation. Front Endocrinol (Lausanne). 2018;9. Available from: https://www.frontiersin.org/article/10.3389/fendo.2018.00402/full. Accessed 3 Aug 2018. 10.3389/fendo.2018.00402 PMC6085463 30123182

[17] Kozomara A, Birgaoanu M, Griffiths-Jones S (2019). miRBase: from microRNA sequences to function. Nucleic Acids Res.

[18] Brennan S, Keon M, Liu B, Su Z, Saksena NK (2019). Panoramic visualization of circulating microRNAs across neurodegenerative diseases in humans. Mol Neurobiol.

[19] Hebert SS, Horre K, Nicolai L, Papadopoulou AS, Mandemakers W, Silahtaroglu AN (2008). Loss of microRNA cluster miR-29a/b-1 in sporadic Alzheimer’s disease correlates with increased BACE1/β-secretase expression. Proc Natl Acad Sci.

[20] Lei X, Lei L, Zhang Z, Zhang Z, Cheng Y (2015). Downregulated miR-29c correlates with increased BACE1 expression in sporadic Alzheimer’s disease. Int J Clin Exp Pathol.

[21] Long JM, Ray B, Lahiri DK (2014). MicroRNA-339-5p down-regulates protein expression of β-site amyloid precursor protein-cleaving enzyme 1 (BACE1) in human primary brain cultures and is reduced in brain tissue specimens of Alzheimer disease subjects. J Biol Chem.

[22] Wang W-X, Huang Q, Hu Y, Stromberg AJ, Nelson PT (2011). Patterns of microRNA expression in normal and early Alzheimer’s disease human temporal cortex: white matter versus gray matter. Acta Neuropathol.

[23] Schipper HM, Maes OC, Chertkow HM, Wang E (2007). MicroRNA expression in Alzheimer blood mononuclear cells. Gene Regul Syst Bio.

[24] Satoh J (2012). Molecular network of microRNA targets in Alzheimer’s disease brains. Exp Neurol.

[25] Cheng L, Quek CYJ, Sun X, Bellingham SA, Hill AF. The detection of microRNA associated with Alzheimer’s disease in biological fluids using next-generation sequencing technologies. Front Genet. 2013;4. Available from: https://www.frontiersin.org/articles/10.3389/fgene.2013.00150/full. Accessed 8 Aug 2013. 10.3389/fgene.2013.00150 PMC3737441 23964286

[26] Hu G, Drescher KM, Chen X-M. Exosomal miRNAs: biological properties and therapeutic potential. Front Genet. 2012;3. Available from: https://www.frontiersin.org/articles/10.3389/fgene.2012.00056/full. Accessed 20 Apr 2012. 10.3389/fgene.2012.00056 PMC3330238 22529849

[27] Kiko T, Nakagawa K, Tsuduki T, Furukawa K, Arai H, Miyazawa T (2014). MicroRNAs in plasma and cerebrospinal fluid as potential markers for Alzheimer’s disease. J Alzheimers Dis.

[28] Geekiyanage H, Jicha GA, Nelson PT, Chan C (2012). Blood serum miRNA: Non-invasive biomarkers for Alzheimer’s disease. Exp Neurol.

[29] Tan L, Yu J-T, Liu Q-Y, Tan M-S, Zhang W, Hu N (2014). Circulating miR-125b as a biomarker of Alzheimer’s disease. J Neurol Sci.

[30] Denk J, Oberhauser F, Kornhuber J, Wiltfang J, Fassbender K, Schroeter ML (2018). Specific serum and CSF microRNA profiles distinguish sporadic behavioural variant of frontotemporal dementia compared with Alzheimer patients and cognitively healthy controls. PLoS One.

[31] Wu Y, Xu J, Xu J, Cheng J, Jiao D, Zhou C (2017). Lower serum levels of miR-29c-3p and miR-19b-3p as biomarkers for Alzheimer’s disease. Tohoku J Exp Med.

[32] Wu HZY, Ong KL, Seeher K, Armstrong NJ, Thalamuthu A, Brodaty H (2015). Circulating microRNAs as biomarkers of Alzheimer’s disease: a systematic review. J Alzheimers Dis.

[33] Zhao Y, Bhattacharjee S, Dua P, Alexandrov PN, Lukiw WJ. microRNA-based biomarkers and the diagnosis of Alzheimer’s disease. Front Neurol. 2015;6. Available from: https://www.frontiersin.org/articles/10.3389/fneur.2015.00162/full. Accessed 13 July 2015. 10.3389/fneur.2015.00162 PMC4499702 26217305

[34] Takousis P, Sadlon A, Schulz J, Wohlers I, Dobricic V, Middleton L (2019). Differential expression of microRNAs in Alzheimer’s disease brain, blood, and cerebrospinal fluid. Alzheimers Dement.

[35] Hu Y-B, Li C-B, Song N, Zou Y, Chen S-D, Ren R-J, et al. Diagnostic value of microRNA for Alzheimer’s disease: a systematic review and meta-analysis. Front Aging Neurosci. 2016;8. Available from: https://www.frontiersin.org/articles/10.3389/fnagi.2016.00013/full. Accessed 9 Feb 2016. 10.3389/fnagi.2016.00013 PMC4746262 26903857

[36] Zhang Y-H, Bai S-F, Yan J-Q (2019). Blood circulating miRNAs as biomarkers of Alzheimer’s disease: a systematic review and meta-analysis. Biomark Med.

[37] Bloudek LM, Spackman DE, Blankenburg M, Sullivan SD (2011). Review and meta-analysis of biomarkers and diagnostic imaging in Alzheimer’s disease. J Alzheimers Dis.

[38] Lugli G, Cohen AM, Bennett DA, Shah RC, Fields CJ, Hernandez AG (2015). Plasma exosomal miRNAs in persons with and without Alzheimer disease: altered expression and prospects for biomarkers. PLoS One.

[39] Ludwig N, Fehlmann T, Kern F, Gogol M, Maetzler W, Deutscher S (2019). Machine learning to detect Alzheimer’s disease from circulating non-coding RNAs. Genomics Proteomics Bioinformatics.

[40] Zhao X, Kang J, Svetnik V, Warden D, Wilcock G, David Smith A (2020). A machine learning approach to identify a circulating microRNA signature for Alzheimer disease. J Appl Lab Med.

[41] Zhao Y, Chen X, Yin J (2019). Adaptive boosting-based computational model for predicting potential miRNA-disease associations. Bioinformatics.

[42] Huang Z, Shi J, Gao Y, Cui C, Zhang S, Li J (2019). HMDD v3.0: a database for experimentally supported human microRNA–disease associations. Nucleic Acids Res.

[43] Moher D, Liberati A, Tetzlaff J, Altman DG (2009). Preferred Reporting Items for Systematic Reviews and Meta-Analyses: the PRISMA Statement. J Clin Epidemiol.

[44] Haunsberger SJ, Connolly NMC, Prehn JHM. miRNAmeConverter: an R/bioconductor package for translating mature miRNA names to different miRBase versions. Bioinformatics. 2016;btw660. Available from: https://academic.oup.com/bioinformatics/article-lookup/doi/10.1093/bioinformatics/btw660. Accessed 15 Feb 2017. 10.1093/bioinformatics/btw660 27797767

[45] Viechtbauer W. Conducting meta-analyses in R with the metafor Package. J Stat Softw. 2010;36 Available from: http://www.jstatsoft.org/v36/i03/. Accessed 5 Aug 2010.

[46] Yu G, Wang L-G, Yan G-R, He Q-Y (2015). DOSE: an R/Bioconductor package for disease ontology semantic and enrichment analysis. Bioinformatics..

[47] Li J, Zhang S, Wan Y, Zhao Y, Shi J, Zhou Y (2019). MISIM v2.0: a web server for inferring microRNA functional similarity based on microRNA-disease associations. Nucleic Acids Res.

[48] Ru Y, Kechris KJ, Tabakoff B, Hoffman P, Radcliffe RA, Bowler R (2014). The multiMiR R package and database: integration of microRNA–target interactions along with their disease and drug associations. Nucleic Acids Res.

[49] Huang HY, Lin YCD, Li J, Huang KY, Shrestha S, Hong HC, et al. miRTarBase 2020: updates to the experimentally validated microRNA–target interaction database. Nucleic Acids Res. 2019. Available from: https://academic.oup.com/nar/advance-article/doi/10.1093/nar/gkz896/5606625. Accessed 8 Jan 2020. 10.1093/nar/gkz896 PMC7145596 31647101

[50] Yu G, Wang L-G, Han Y, He Q-Y (2012). clusterProfiler: an R Package for comparing biological themes among gene clusters. Omi A J Integr Biol.

[51] Kanehisa M, Furumichi M, Tanabe M, Sato Y, Morishima K (2017). KEGG: new perspectives on genomes, pathways, diseases and drugs. Nucleic Acids Res.

[52] Szklarczyk D, Gable AL, Lyon D, Junge A, Wyder S, Huerta-Cepas J (2019). STRING v11: protein-protein association networks with increased coverage, supporting functional discovery in genome-wide experimental datasets. Nucleic Acids Res.

[53] Bustin SA, Benes V, Garson JA, Hellemans J, Huggett J, Kubista M (2009). The MIQE guidelines: minimum information for publication of quantitative real-time PCR experiments. Clin Chem.

[54] Kenny A, McArdle H, Calero M, Rabano A, Madden SF, Adamson K (2019). Elevated plasma microRNA-206 levels predict cognitive decline and progression to dementia from mild cognitive impairment. Biomolecules..

[55] Barbagallo C, Mostile G, Baglieri G, Giunta F, Luca A, Raciti L (2020). Specific signatures of serum miRNAs as potential biomarkers to discriminate clinically similar neurodegenerative and vascular-related diseases. Cell Mol Neurobiol.

[56] Gámez-Valero A, Campdelacreu J, Vilas D, Ispierto L, Reñé R, Álvarez R (2019). Exploratory study on microRNA profiles from plasma-derived extracellular vesicles in Alzheimer’s disease and dementia with Lewy bodies. Transl Neurodegener.

[57] Yang Q, Zhao Q, Yin Y. miR-133b is a potential diagnostic biomarker for Alzheimer’s disease and has a neuroprotective role. Exp Ther Med. 2019. Available from: http://www.spandidos-publications.com/10.3892/etm.2019.7855. Accessed 5 Aug 2019. 10.3892/etm.2019.7855 PMC6755445 31572518

[58] Wang J, Chen C, Zhang Y. An investigation of microRNA-103 and microRNA-107 as potential blood-based biomarkers for disease risk and progression of Alzheimer’s disease. J Clin Lab Anal. 2020;34. Available from: https://onlinelibrary.wiley.com/doi/abs/10.1002/jcla.23006. Accessed 16 Aug 2019. 10.1002/jcla.23006 PMC6977154 31420923

[59] Siedlecki-Wullich D, Català-Solsona J, Fábregas C, Hernández I, Clarimon J, Lleó A (2019). Altered microRNAs related to synaptic function as potential plasma biomarkers for Alzheimer’s disease. Alzheimers Res Ther.

[60] Zhou Q, Luo L, Wang X, Li X. Relationship between single nucleotide polymorphisms in the 3′UTR of amyloid precursor protein and risk of Alzheimer’s disease and its mechanism. Biosci Rep. 2019;39. Available from: https://portlandpress.com/bioscirep/article/doi/10.1042/BSR20182485/219091/Relationship-between-single-nucleotide. Accessed 2 May 2019. 10.1042/BSR20182485 PMC6499457 30914454

[61] Geng L, Zhang T, Liu W, Chen Y (2018). Inhibition of miR-128 Abates Aβ-mediated cytotoxicity by targeting PPAR-γ via NF-κB inactivation in primary mouse cortical neurons and Neuro2a cells. Yonsei Med J.

[62] Dias IHK, Brown CL, Shabir K, Polidori MC, Griffiths HR (2018). miRNA 933 expression by endothelial cells is increased by 27-hydroxycholesterol and is more prevalent in plasma from dementia patients. J Alzheimers Dis.

[63] Wang Z, Qin W, Wei CB, Tang Y, Zhao LN, Jin HM (2018). The microRNA-1908 up-regulation in the peripheral blood cells impairs amyloid clearance by targeting ApoE. Int J Geriatr Psychiatry.

[64] Yang TT, Liu CG, Gao SC, Zhang Y, WangP C (2018). The serum exosome derived microRNA-135a, -193b, and -384 were potential Alzheimer’s disease biomarkers. Biomed Environ Sci.

[65] Manzine PR, Pelucchi S, Horst MA, Vale FAC, Pavarini SCI, Audano M (2017). microRNA 221 targets ADAM10 mRNA and is downregulated in Alzheimer’s disease. J Alzheimers Dis.

[66] Guo R, Fan G, Zhang J, Wu C, Du Y, Ye H (2017). A 9-microRNA signature in serum serves as a noninvasive biomarker in early diagnosis of Alzheimer’s disease. J Alzheimers Dis.

[67] Kumar S, Vijayan M, Reddy PH (2017). MicroRNA-455-3p as a potential peripheral biomarker for Alzheimer’s disease. Hum Mol Genet.

[68] Zeng Q, Zou L, Qian L, Zhou F, Nie H, Yu S (2017). Expression of microRNA-222 in serum of patients with Alzheimer’s disease. Mol Med Rep.

[69] Nagaraj S, Laskowska-Kaszub K, Dębski KJ, Wojsiat J, Dąbrowski M, Gabryelewicz T (2017). Profile of 6 microRNA in blood plasma distinguish early stage Alzheimer’s disease patients from non-demented subjects. Oncotarget.

[70] Hara N, Kikuchi M, Miyashita A, Hatsuta H, Saito Y, Kasuga K (2017). Serum microRNA miR-501-3p as a potential biomarker related to the progression of Alzheimer’s disease. Acta Neuropathol Commun.

[71] Li W, Li X, Xin X, Kan P-C, Yan Y (2016). MicroRNA-613 regulates the expression of brain-derived neurotrophic factor in Alzheimer’s disease. Biosci Trends.

[72] Yılmaz ŞG, Erdal ME, Özge AA, Sungur MA (2016). Can peripheral microRNA expression data serve as epigenomic (upstream) biomarkers of Alzheimer’s disease?. Omi A J Integr Biol.

[73] Zhang Y, Xing H, Guo S, Zheng Z, Wang H, Xu D (2016). MicroRNA-135b has a neuroprotective role via targeting of β-site APP-cleaving enzyme 1. Exp Ther Med.

[74] Xing H, Guo S, Zhang Y, Zheng Z, Wang H (2016). Upregulation of microRNA-206 enhances lipopolysaccharide-induced inflammation and release of amyloid-β by targeting insulin-like growth factor 1 in microglia. Mol Med Rep.

[75] Guedes JR, Santana I, Cunha C, Duro D, Almeida MR, Cardoso AM (2016). MicroRNA deregulation and chemotaxis and phagocytosis impairment in Alzheimer’s disease. Alzheimers Dement Diagn Assess Dis Monit.

[76] Jia L-H, Liu Y-N (2016). Downregulated serum miR-223 servers as biomarker in Alzheimer’s disease. Cell Biochem Funct.

[77] Ragusa M, Bosco P, Tamburello L, Barbagallo C, Condorelli AG, Tornitore M, et al. miRNAs plasma profiles in vascular dementia: biomolecular data and biomedical implications. Front Cell Neurosci. 2016;10. Available from: http://journal.frontiersin.org/article/10.3389/fncel.2016.00051. Accessed 1 Mar 2016. 10.3389/fncel.2016.00051 PMC4771726 26973465

[78] Ren R-J, Zhang Y-F, Dammer EB, Zhou Y, Wang L, Liu X-H (2016). Peripheral blood microRNA expression profiles in Alzheimer’s disease: screening, validation, association with clinical phenotype and implications for molecular mechanism. Mol Neurobiol.

[79] Dong H, Li J, Huang L, Chen X, Li D, Wang T (2015). Serum microRNA profiles serve as novel biomarkers for the diagnosis of Alzheimer’s disease. Dis Markers.

[80] Yang G, Song Y, Zhou X, Deng Y, Liu T, Weng G (2015). MicroRNA-29c targets β-site amyloid precursor protein-cleaving enzyme 1 and has a neuroprotective role in vitro and in vivo. Mol Med Rep.

[81] Wang T, Chen K, Li H, Dong S, Su N, Liu Y (2015). The feasibility of utilizing plasma <em>MiRNA107</em> and < em>BACE1</em> messenger RNA gene expression for clinical diagnosis of amnestic mild cognitive impairment. J Clin Psychiatry.

[82] Zhu Y, Li C, Sun A, Wang Y, Zhou S (2015). Quantification of microRNA-210 in the cerebrospinal fluid and serum: implications for Alzheimer’s disease. Exp Ther Med.

[83] Liu C, Wang J, Li L, Xue L, Zhang Y, Wang P (2014). MicroRNA-135a and -200b, potential biomarkers for Alzheimer′s disease, regulate β secretase and amyloid precursor protein. Brain Res.

[84] Liu C-G, Wang J-L, Li L, Wang P-C (2014). MicroRNA-384 regulates both amyloid precursor protein and β-secretase expression and is a potential biomarker for Alzheimer’s disease. Int J Mol Med.

[85] Tan L, Yu J-T, Tan M-S, Liu Q-Y, Wang H-F, Zhang W (2014). Genome-wide serum microRNA expression profiling identifies serum biomarkers for Alzheimer’s disease. J Alzheimers Dis.

[86] Bhatnagar S, Chertkow H, Schipper HM, Yuan Z, Shetty V, Jenkins S, et al. Increased microRNA-34c abundance in Alzheimer’s disease circulating blood plasma. Front Mol Neurosci. 2014;7. Available from: https://www.frontiersin.org/articles/10.3389/fnmol.2014.00002/full. Accessed 4 Feb 2014. 10.3389/fnmol.2014.00002 PMC3912349 24550773

[87] Tiribuzi R, Crispoltoni L, Porcellati S, DiLullo M, Florenzano F, Pirro M (2014). miR128 up-regulation correlates with impaired amyloid β(1-42) degradation in monocytes from patients with sporadic Alzheimer’s disease. Neurobiol Aging.

[88] Kumar P, Dezso Z, MacKenzie C, Oestreicher J, Agoulnik S, Byrne M (2013). Circulating miRNA biomarkers for Alzheimer’s disease. PLoS One.

[89] Leidinger P, Backes C, Deutscher S, Schmitt K, Mueller SC, Frese K (2013). A blood based 12-miRNA signature of Alzheimer disease patients. Genome Biol.

[90] Villa C, Ridolfi E, Fenoglio C, Ghezzi L, Vimercati R, Clerici F (2013). Expression of the transcription Factor Sp1 and its regulatory hsa-miR-29b in peripheral blood mononuclear cells from patients with Alzheimer’s disease. J Alzheimers Dis.

[91] Cao F, Liu Z, Sun G (2020). Diagnostic value of miR-193a-3p in Alzheimer’s disease and miR-193a-3p attenuates amyloid-β induced neurotoxicity by targeting PTEN. Exp Gerontol.

[92] Cha DJ, Mengel D, Mustapic M, Liu W, Selkoe DJ, Kapogiannis D (2019). MiR-212 and miR-132 are downregulated in neurally derived plasma exosomes of Alzheimer’s patients. Front Neurosci.

[93] Jiao Y, Kong L, Yao Y, Li S, Tao Z, Yan Y (2016). Osthole decreases beta amyloid levels through up-regulation of miR-107 in Alzheimer’s disease. Neuropharmacology..

[94] Moncini S, Salvi A, Zuccotti P, Viero G, Quattrone A, Barlati S (2011). The role of miR-103 and miR-107 in regulation of CDK5R1 expression and in cellular migration. PLoS One.

[95] Hébert SS, Horré K, Nicolaï L, Bergmans B, Papadopoulou AS, Delacourte A (2009). MicroRNA regulation of Alzheimer’s amyloid precursor protein expression. Neurobiol Dis.

[96] Zhang Y, Wang J, Liu X, Li J, Fan S (2020). MicroRNA miR-103a-3p targets NPAS3 to regulate progression of Alzheimer’s disease. Trop J Pharm Res.

[97] Manzano-Crespo M, Atienza M, Cantero JL (2019). Lower serum expression of miR-181c-5p is associated with increased plasma levels of amyloid-beta 1-40 and cerebral vulnerability in normal aging. Transl Neurodegener.

[98] Guo W-G, Zhang Y, Ge D, Zhang Y-X, Lu C-L, Wang Q (2013). Bioinformatics analyses combined microarray identify the desregulated microRNAs in lung cancer. Eur Rev Med Pharmacol Sci.

[99] Zhuang L, Xu L, Wang P, Meng Z (2015). Serum miR-128-2 serves as a prognostic marker for patients with hepatocellular carcinoma. PLoS One.

[100] Chen X, Xu Y, Liao X, Liao R, Zhang L, Niu K (2016). Plasma miRNAs in predicting radiosensitivity in non-small cell lung cancer. Tumor Biol.

[101] Yanaihara N, Caplen N, Bowman E, Seike M, Kumamoto K, Yi M (2006). Unique microRNA molecular profiles in lung cancer diagnosis and prognosis. Cancer Cell.

[102] Shi W, Dong F, Jiang Y, Lu L, Wang C, Tan J (2019). Construction of prognostic microRNA signature for human invasive breast cancer by integrated analysis. Onco Targets Ther.

[103] Zhou X, Zhang Z, Liang X (2020). Regulatory network analysis to reveal important miRNAs and genes in non-small cell lung cancer. Cell J.

[104] Kirschner MB, Cheng YY, Badrian B, Kao SC, Creaney J, Edelman JJB (2012). Increased circulating miR-625-3p: a potential biomarker for patients with malignant pleural mesothelioma. J Thorac Oncol.

[105] Zhou Y, Xu Z, Yu Y, Cao J, Qiao Y, Qiao H (2019). Comprehensive analysis of the lncRNA-associated ceRNA network identifies neuroinflammation biomarkers for Alzheimer’s disease. Mol Omi.

[106] Mei L, He M, Zhang C, Miao J, Wen Q, Liu X (2019). Paeonol attenuates inflammation by targeting HMGB1 through upregulating miR-339-5p. Sci Rep.

[107] Andersson Å, Covacu R, Sunnemark D, Danilov AI, Dal Bianco A, Khademi M (2008). Pivotal advance: HMGB1 expression in active lesions of human and experimental multiple sclerosis. J Leukoc Biol.

[108] Zhang Y, Wei G, Di Z, Zhao Q (2014). miR-339-5p inhibits alcohol-induced brain inflammation through regulating NF-κB pathway. Biochem Biophys Res Commun.

[109] Piñeiro-Hermida S, López IP, Alfaro-Arnedo E, Torrens R, Iñiguez M, Alvarez-Erviti L (2017). IGF1R deficiency attenuates acute inflammatory response in a bleomycin-induced lung injury mouse model. Sci Rep.

[110] Xu H, Liu C, Zhang Y, Guo X, Liu Z, Luo Z (2014). Let-7b-5p regulates proliferation and apoptosis in multiple myeloma by targeting IGF1R. Acta Biochim Biophys Sin Shanghai.

[111] Jin H, Kim T-J, Choi J-H, Kim M-J, Cho Y-N, Nam K-I (2014). MicroRNA-155 as a proinflammatory regulator via SHIP-1 down-regulation in acute gouty arthritis. Arthritis Res Ther.

[112] Qian F-H, Deng X, Zhuang Q-X, Wei B, Zheng D-D. miR-625-5p suppresses inflammatory responses by targeting AKT2 in human bronchial epithelial cells. Mol Med Rep. 2019. Available from: http://www.spandidos-publications.com/10.3892/mmr.2019.9817. Accessed 3 Jan 2019. 10.3892/mmr.2019.9817 30628701

[113] Wang X, Jin H, Jiang S, Xu Y (2018). MicroRNA-495 inhibits the high glucose-induced inflammation, differentiation and extracellular matrix accumulation of cardiac fibroblasts through downregulation of NOD1. Cell Mol Biol Lett.

[114] Lin X, Lin Q. MiRNA-495-3p attenuates TNF-α induced apoptosis and inflammation in human nucleus pulposus cells by targeting IL5RA. Inflammation. 2020. Available from: http://link.springer.com/10.1007/s10753-020-01254-5. Accessed 22 May 2020. 10.1007/s10753-020-01254-5 32445070

[115] Cho KJ, Song J, Oh Y, Lee JE (2015). MicroRNA-Let-7a regulates the function of microglia in inflammation. Mol Cell Neurosci.

[116] Caggiano R, Cattaneo F, Moltedo O, Esposito G, Perrino C, Trimarco B (2017). miR-128 is implicated in stress responses by targeting MAFG in skeletal muscle cells. Oxidative Med Cell Longev.

[117] Tian T, Zhou Y, Feng X, Ye S, Wang H, Wu W (2016). MicroRNA-16 is putatively involved in the NF-κB pathway regulation in ulcerative colitis through adenosine A2a receptor (A2aAR) mRNA targeting. Sci Rep.

[118] Lu Q, Ma Z, Ding Y, Bedarida T, Chen L, Xie Z (2019). Circulating miR-103a-3p contributes to angiotensin II-induced renal inflammation and fibrosis via a SNRK/NF-κB/p65 regulatory axis. Nat Commun.

[119] Drenth H, Zuidema SU, Krijnen WP, Bautmans I, van der Schans C, Hobbelen H (2017). Advanced glycation end-products are associated with the presence and severity of paratonia in early stage Alzheimer disease. J Am Med Dir Assoc.

[120] Miller MC, Tavares R, Johanson CE, Hovanesian V, Donahue JE, Gonzalez L (2008). Hippocampal RAGE immunoreactivity in early and advanced Alzheimer’s disease. Brain Res.

[121] Wan W, Cao L, Liu L, Zhang C, Kalionis B, Tai X (2015). Aβ 1-42 oligomer-induced leakage in an in vitro blood-brain barrier model is associated with up-regulation of RAGE and metalloproteinases, and down-regulation of tight junction scaffold proteins. J Neurochem.

[122] Tobon-Velasco J, Cuevas E, Torres-Ramos M (2014). Receptor for AGEs (RAGE) as mediator of NF-kB pathway activation in neuroinflammation and oxidative stress. CNS Neurol Disord Drug Targets.

[123] Sastre M, Dewachter I, Rossner S, Bogdanovic N, Rosen E, Borghgraef P (2006). Nonsteroidal anti-inflammatory drugs repress β-secretase gene promoter activity by the activation of PPAR. Proc Natl Acad Sci.

[124] Yamamoto M, Kiyota T, Horiba M, Buescher JL, Walsh SM, Gendelman HE (2007). Interferon-γ and tumor necrosis factor-α regulate amyloid-β plaque deposition and β-secretase expression in Swedish mutant APP transgenic mice. Am J Pathol.

[125] Faden AI, Loane DJ (2015). Chronic neurodegeneration after traumatic brain injury: Alzheimer disease, chronic traumatic encephalopathy, or persistent neuroinflammation?. Neurotherapeutics..

[126] Norambuena A, Wallrabe H, McMahon L, Silva A, Swanson E, Khan SS (2017). mTOR and neuronal cell cycle reentry: how impaired brain insulin signaling promotes Alzheimer’s disease. Alzheimers Dement.

[127] Seward ME, Swanson E, Norambuena A, Reimann A, Cochran JN, Li R (2013). Amyloid-β signals through tau to drive ectopic neuronal cell cycle re-entry in Alzheimer’s disease. J Cell Sci.

[128] Barrio-Alonso E, Fontana B, Valero M, Frade JM. Pathological aspects of neuronal hyperploidization in Alzheimer’s disease evidenced by computer simulation. Front Genet. 2020;11. Available from: https://www.frontiersin.org/article/10.3389/fgene.2020.00287/full. Accessed 27 Mar 2020. 10.3389/fgene.2020.00287 PMC7121139 32292421

[129] Barrio-Alonso E, Hernández-Vivanco A, Walton CC, Perea G, Frade JM (2018). Cell cycle reentry triggers hyperploidization and synaptic dysfunction followed by delayed cell death in differentiated cortical neurons. Sci Rep.

[130] Bell KFS, Hardingham GE (2011). The influence of synaptic activity on neuronal health. Curr Opin Neurobiol.

[131] Musicco M, Adorni F, DiSanto S, Prinelli F, Pettenati C, Caltagirone C (2013). Inverse occurrence of cancer and Alzheimer disease: a population-based incidence study. Neurology..

[132] Boccardi V, Pelini L, Ercolani S, Ruggiero C, Mecocci P (2015). From cellular senescence to Alzheimer’s disease: the role of telomere shortening. Ageing Res Rev.

[133] Flanary BE, Sammons NW, Nguyen C, Walker D, Streit WJ (2007). Evidence that aging and amyloid promote microglial cell senescence. Rejuvenation Res.

[134] Ribezzo F, Shiloh Y, Schumacher B (2016). Systemic DNA damage responses in aging and diseases. Semin Cancer Biol.

[135] Wei Z, Chen X-C, Song Y, Pan X-D, Dai X-M, Zhang J (2016). Amyloid β protein aggravates neuronal senescence and cognitive deficits in 5XFAD mouse model of Alzheimerʼs disease. Chin Med J.

[136] Ardestani A, Lupse B, Maedler K (2018). Hippo signaling: key emerging pathway in cellular and whole-body metabolism. Trends Endocrinol Metab.

[137] Irwin M, Tare M, Singh A, Puli OR, Gogia N, Riccetti M, et al. A positive feedback loop of Hippo- and c-Jun-amino-terminal kinase signaling pathways regulates amyloid-beta-mediated neurodegeneration. Front Cell Dev Biol. 2020;8. Available from: https://www.frontiersin.org/article/10.3389/fcell.2020.00117/full. Accessed 13 Mar 2020. 10.3389/fcell.2020.00117 PMC7082232 32232042

[138] Cao D, Lu H, Lewis TL, Li L (2007). Intake of sucrose-sweetened water induces insulin resistance and exacerbates memory deficits and amyloidosis in a transgenic mouse model of Alzheimer disease. J Biol Chem.

[139] Neumann K, Rojo L, Navarrete L, Farias G, Reyes P, Maccioni R (2008). Insulin resistance and Alzheimers disease: molecular links & clinical implications. Curr Alzheimer Res.

[140] Storz P (2011). Forkhead homeobox type O transcription factors in the responses to oxidative stress. Antioxid Redox Signal.

[141] Yuen SC, Zhu H, Leung S. A systematic bioinformatics workflow with meta-analytics identified potential pathogenic factors of Alzheimer’s disease. Front Neurosci. 2020;14. Available from: https://www.frontiersin.org/article/10.3389/fnins.2020.00209/full. Accessed 3 Mar 2020. 10.3389/fnins.2020.00209 PMC7083177 32231518

[142] Ricciotti E, FitzGerald GA (2011). Prostaglandins and inflammation. Arterioscler Thromb Vasc Biol.

[143] Figueiredo-Pereira ME, Rockwell P, Schmidt-Glenewinkel T, Serrano P. Neuroinflammation and J2 prostaglandins: linking impairment of the ubiquitin-proteasome pathway and mitochondria to neurodegeneration. Front Mol Neurosci. 2015;7. Available from: https://www.frontiersin.org/articles/10.3389/fnmol.2014.00104/full. Accessed 13 Jan 2015. 10.3389/fnmol.2014.00104 PMC4292445 25628533

[144] Chauhan M, Modi PK, Sharma P (2020). Aberrant activation of neuronal cell cycle caused by dysregulation of ubiquitin ligase Itch results in neurodegeneration. Cell Death Dis.

[145] Marini A, Rotblat B, Sbarrato T, Niklison-Chirou MV, Knight JRP, Dudek K (2018). TAp73 contributes to the oxidative stress response by regulating protein synthesis. Proc Natl Acad Sci.

[146] Deyati A, Younesi E, Hofmann-Apitius M, Novac N (2013). Challenges and opportunities for oncology biomarker discovery. Drug Discov Today.

[147] Morris JC, Roe CM, Grant EA, Head D, Storandt M, Goate AM, et al. Pittsburgh Compound B imaging and prediction of progression from cognitive normality to symptomatic Alzheimer disease. Arch Neurol. 2009;66 Available from: http://archneur.jamanetwork.com/article.aspx?doi=10.1001/archneurol.2009.269. Accessed Dec 2009. 10.1001/archneurol.2009.269 PMC2798814 20008650

[148] Forsberg A, Engler H, Almkvist O, Blomquist G, Hagman G, Wall A (2008). PET imaging of amyloid deposition in patients with mild cognitive impairment. Neurobiol Aging.

[149] Furney SJ, Kronenberg D, Simmons A, Güntert A, Dobson RJ, Proitsi P (2011). Combinatorial markers of mild cognitive impairment conversion to Alzheimer’s disease - cytokines and MRI measures together predict disease progression. J Alzheimers Dis.

[150] Wang W-X, Rajeev BW, Stromberg AJ, Ren N, Tang G, Huang Q (2008). The expression of microRNA miR-107 decreases early in Alzheimer’s disease and may accelerate disease progression through regulation of beta-site amyloid precursor protein-cleaving enzyme 1. J Neurosci.

[151] Augustin R, Endres K, Reinhardt S, Kuhn P-H, Lichtenthaler SF, Hansen J (2012). Computational identification and experimental validation of microRNAs binding to the Alzheimer-related gene ADAM10. BMC Med Genet.

[152] Geekiyanage H, Chan C (2011). MicroRNA-137/181c regulates serine palmitoyltransferase and in turn amyloid β, novel targets in sporadic Alzheimer’s disease. J Neurosci.

[153] Hutchison ER, Kawamoto EM, Taub DD, Lal A, Abdelmohsen K, Zhang Y, et al. Evidence for miR-181 involvement in neuroinflammatory responses of astrocytes. Glia [Internet]. 2013;61:1018–28. Available from: http://doi.wiley.com/10.1002/glia.22483. 10.1002/glia.22483 PMC4624280 23650073

[154] Ji Q, Wang X, Cai J, Du X, Sun H, Zhang N (2019). MiR-22-3p regulates amyloid β deposit in mice model of Alzheimer’s disease by targeting mitogen-activated protein kinase 14. Curr Neurovasc Res.

[155] Zong Y, Wang H, Dong W, Quan X, Zhu H, Xu Y (2011). miR-29c regulates BACE1 protein expression. Brain Res.

[156] Madadi S, Schwarzenbach H, Saidijam M, Mahjub R, Soleimani M (2019). Potential microRNA-related targets in clearance pathways of amyloid-β: novel therapeutic approach for the treatment of Alzheimer’s disease. Cell Biosci.

[157] Zovoilis A, Agbemenyah HY, Agis-Balboa RC, Stilling RM, Edbauer D, Rao P (2011). microRNA-34c is a novel target to treat dementias. EMBO J.

[158] Hu S, Wang H, Chen K, Cheng P, Gao S, Liu J (2015). MicroRNA-34c downregulation ameliorates amyloid-β-induced synaptic failure and memory deficits by targeting VAMP2. J Alzheimers Dis.

[159] Wang L, Liu J, Wang Q, Jiang H, Zeng L, Li Z (2019). MicroRNA-200a-3p mediates neuroprotection in Alzheimer-related deficits and attenuates amyloid-beta overproduction and tau hyperphosphorylation via coregulating BACE1 and PRKACB. Front Pharmacol.

[160] Hansen KF, Sakamoto K, Aten S, Snider KH, Loeser J, Hesse AM (2016). Targeted deletion of miR-132/-212 impairs memory and alters the hippocampal transcriptome. Learn Mem.

[161] Smith PY, Hernandez-Rapp J, Jolivette F, Lecours C, Bisht K, Goupil C (2015). miR-132/212 deficiency impairs tau metabolism and promotes pathological aggregation in vivo. Hum Mol Genet.

[162] Salta E, Sierksma A, Vanden Eynden E, DeStrooper B (2016). miR-132 loss de-represses ITPKB and aggravates amyloid and TAU pathology in Alzheimer’s brain. EMBO Mol Med.

[163] Hernandez-Rapp J, Rainone S, Goupil C, Dorval V, Smith PY, Saint-Pierre M (2016). microRNA-132/212 deficiency enhances Aβ production and senile plaque deposition in Alzheimer’s disease triple transgenic mice. Sci Rep.

[164] El Fatimy R, Li S, Chen Z, Mushannen T, Gongala S, Wei Z (2018). MicroRNA-132 provides neuroprotection for tauopathies via multiple signaling pathways. Acta Neuropathol.

[165] Liu W, Zhao J, Lu G (2016). miR-106b inhibits tau phosphorylation at Tyr18 by targeting Fyn in a model of Alzheimer’s disease. Biochem Biophys Res Commun.

[166] Siedlecki-Wullich D, Miñano-Molina AJ, Rodríguez-Álvarez J. microRNAs as early biomarkers of Alzheimer’s disease: a synaptic perspective. Cells. 2021;10. Available from: http://www.ncbi.nlm.nih.gov/pubmed/33435363. Accessed Jan 2021. 10.3390/cells10010113 PMC7827653 33435363

[167] Haidar M, Rchiad Z, Ansari HR, Ben-Rached F, Tajeri S, Latre De Late P (2018). miR-126-5p by direct targeting of JNK-interacting protein-2 (JIP-2) plays a key role in Theileria-infected macrophage virulence. PLoS Pathog.

[168] Fairchild CLA, Cheema SK, Wong J, Hino K, Simó S, La Torre A (2019). Let-7 regulates cell cycle dynamics in the developing cerebral cortex and retina. Sci Rep.

[169] Buonfiglioli A, Efe IE, Guneykaya D, Ivanov A, Huang Y, Orlowski E (2019). let-7 MicroRNAs regulate microglial function and suppress glioma growth through Toll-like receptor 7. Cell Rep.

[170] Cogswell JP, Ward J, Taylor IA, Waters M, Shi Y, Cannon B (2008). Identification of miRNA changes in Alzheimer’s disease brain and CSF yields putative biomarkers and insights into disease pathways. J Alzheimers Dis.

[171] Arendt T, Brückner MK, Mosch B, Lösche A (2010). Selective cell death of hyperploid neurons in Alzheimer’s disease. Am J Pathol.

[172] Herrup K, Yang Y (2007). Cell cycle regulation in the postmitotic neuron: oxymoron or new biology?. Nat Rev Neurosci.

[173] van Leeuwen LAG, Hoozemans JJM (2015). Physiological and pathophysiological functions of cell cycle proteins in post-mitotic neurons: implications for Alzheimer’s disease. Acta Neuropathol.

[174] Yang Y, Mufson EJ, Herrup K (2003). Neuronal cell death is preceded by cell cycle events at all stages of Alzheimer’s disease. J Neurosci.

[175] Varvel NH, Bhaskar K, Patil AR, Pimplikar SW, Herrup K, Lamb BT (2008). A oligomers induce neuronal cell cycle events in Alzheimer’s disease. J Neurosci.

[176] Kodis EJ, Choi S, Swanson E, Ferreira G, Bloom GS (2018). N-methyl-D-aspartate receptor-mediated calcium influx connects amyloid-β oligomers to ectopic neuronal cell cycle reentry in Alzheimer’s disease. Alzheimers Dement.

[177] Williamson R, Usardi A, Hanger DP, Anderton BH (2008). Membrane-bound β-amyloid oligomers are recruited into lipid rafts by a fyn-dependent mechanism. FASEB J.

[178] Zhao W, DeFelice FG, Fernandez S, Chen H, Lambert MP, Quon MJ (2008). Amyloid beta oligomers induce impairment of neuronal insulin receptors. FASEB J.

[179] dela Monte SM (2014). Type 3 diabetes is sporadic Alzheimer′s disease: mini-review. Eur Neuropsychopharmacol.

[180] Steen E, Terry BM, Rivera EJ, Cannon JL, Neely TR, Tavares R (2005). Impaired insulin and insulin-like growth factor expression and signaling mechanisms in Alzheimer’s disease--is this type 3 diabetes?. J Alzheimers Dis.

[181] Bedse G, DiDomenico F, Serviddio G, Cassano T. Aberrant insulin signaling in Alzheimer’s disease: current knowledge. Front Neurosci. 2015;9. Available from: https://www.frontiersin.org/articles/10.3389/fnins.2015.00204/full. Accessed 16 June 2015. 10.3389/fnins.2015.00204 PMC4468388 26136647

[182] Iacono D, O’Brien R, Resnick SM, Zonderman AB, Pletnikova O, Rudow G (2008). Neuronal hypertrophy in asymptomatic Alzheimer disease. J Neuropathol Exp Neurol.

[183] Iacono D, Markesbery WR, Gross M, Pletnikova O, Rudow G, Zandi P (2009). The Nun study: clinically silent AD, neuronal hypertrophy, and linguistic skills in early life. Neurology..

[184] Raina AK, Hochman A, Zhu X, Rottkamp CA, Nunomura A, Siedlak SL (2001). Abortive apoptosis in Alzheimer’s disease. Acta Neuropathol.

[185] Ginsberg SD, Alldred MJ, Counts SE, Cataldo AM, Neve RL, Jiang Y (2010). Microarray analysis of hippocampal CA1 neurons implicates early endosomal dysfunction during Alzheimer’s disease progression. Biol Psychiatry.

[186] Peng S, Wuu J, Mufson EJ, Fahnestock M (2004). Increased proNGF levels in subjects with mild cognitive impairment and mild Alzheimer disease. J Neuropathol Exp Neurol.

[187] Yuan Z, Becker EBE, Merlo P, Yamada T, DiBacco S, Konishi Y (2008). Activation of FOXO1 by Cdk1 in cycling cells and postmitotic neurons. Science..

[188] Khan SS, Bloom GS. Tau: the center of a signaling nexus in Alzheimer’s disease. Front Neurosci. 2016;10. Available from: https://www.frontiersin.org/articles/10.3389/fnins.2016.00031/full. Accessed 9 Feb 2016. 10.3389/fnins.2016.00031 PMC4746348 26903798

[189] Zhang B, Gaiteri C, Bodea LG, Wang Z, McElwee J, Podtelezhnikov AA (2013). Integrated systems approach identifies genetic nodes and networks in late-onset Alzheimer’s disease. Cell..

[190] Edison P, Archer HA, Gerhard A, Hinz R, Pavese N, Turkheimer FE (2008). Microglia, amyloid, and cognition in Alzheimer’s disease: An [11C](R)PK11195-PET and [11C]PIB-PET study. Neurobiol Dis.

[191] Belkhelfa M, Rafa H, Medjeber O, Arroul-Lammali A, Behairi N, Abada-Bendib M (2014). IFN-γ and TNF-α are involved during Alzheimer disease progression and correlate with nitric oxide production: a study in Algerian patients. J Interf Cytokine Res.

[192] Eikelenboom P, van Exel E, Hoozemans JJM, Veerhuis R, Rozemuller AJM (2010). vanGoolWA. Neuroinflammation – an early event in both the history and pathogenesis of Alzheimer’s disease. Neurodegener Dis.

[193] Thériault P, ElAli A, Rivest S (2015). The dynamics of monocytes and microglia in Alzheimer’s disease. Alzheimers Res Ther.

[194] Bertram L, Lange C, Mullin K, Parkinson M, Hsiao M, Hogan MF (2008). Genome-wide association analysis reveals putative Alzheimer’s disease susceptibility loci in addition to APOE. Am J Hum Genet.

[195] Lambert J-C, Heath S, Even G, Campion D, Sleegers K, Hiltunen M (2009). Genome-wide association study identifies variants at CLU and CR1 associated with Alzheimer’s disease. Nat Genet.

[196] Naj AC, Jun G, Beecham GW, Wang L-S, Vardarajan BN, Buros J (2011). Common variants at MS4A4/MS4A6E, CD2AP, CD33 and EPHA1 are associated with late-onset Alzheimer’s disease. Nat Genet.

[197] Hollingworth P, Harold D, Sims R, Gerrish A, Lambert J-C, Carrasquillo MM (2011). Common variants at ABCA7, MS4A6A/MS4A4E, EPHA1, CD33 and CD2AP are associated with Alzheimer’s disease. Nat Genet.

[198] Lunnon K, Smith R, Hannon E, DeJager PL, Srivastava G, Volta M (2014). Methylomic profiling implicates cortical deregulation of ANK1 in Alzheimer’s disease. Nat Neurosci.

[199] Mastroeni D, Sekar S, Nolz J, Delvaux E, Lunnon K, Mill J (2017). ANK1 is up-regulated in laser captured microglia in Alzheimer’s brain; the importance of addressing cellular heterogeneity. PLoS One.

[200] Passamonti L, Tsvetanov KA, Jones PS, Bevan-Jones WR, Arnold R, Borchert RJ (2019). Neuroinflammation and functional connectivity in Alzheimer’s disease: interactive influences on cognitive performance. J Neurosci.

[201] Yirmiya R, Goshen I (2011). Immune modulation of learning, memory, neural plasticity and neurogenesis. Brain Behav Immun.

[202] Hong S, Beja-Glasser VF, Nfonoyim BM, Frouin A, Li S, Ramakrishnan S (2016). Complement and microglia mediate early synapse loss in Alzheimer mouse models. Science..

[203] Martin BK, Szekely C, Brandt J, Piantadosi S, Breitner JCS, Craft S (2008). Cognitive function over time in the Alzheimer’s Disease Anti-inflammatory Prevention Trial (ADAPT). Arch Neurol.

[204] Breitner JC, Baker LD, Montine TJ, Meinert CL, Lyketsos CG, Ashe KH (2011). Extended results of the Alzheimer’s disease anti-inflammatory prevention trial. Alzheimers Dement.

[205] Wilcock GK, Black SE, Hendrix SB, Zavitz KH, Swabb EA, Laughlin MA (2008). Efficacy and safety of tarenflurbil in mild to moderate Alzheimer’s disease: a randomised phase II trial. Lancet Neurol.

[206] Green RC, Schneider LS, Amato DA, Beelen AP, Wilcock G, Swabb EA (2009). Effect of tarenflurbil on cognitive decline and activities of daily living in patients with mild Alzheimer disease: a randomized controlled trial. JAMA.

[207] Muntimadugu E, Dhommati R, Jain A, Challa VGS, Shaheen M, Khan W (2016). Intranasal delivery of nanoparticle encapsulated tarenflurbil: a potential brain targeting strategy for Alzheimer’s disease. Eur J Pharm Sci.

[208] Blandford SN, Galloway DA, Moore CS. The roles of extracellular vesicle microRNAs in the central nervous system. Glia. 2018:2267–78. Available from: https://onlinelibrary.wiley.com/doi/abs/10.1002/glia.23445. Accessed 4 May 2018. 10.1002/glia.23445 29726599

[209] Takeda S, Sato N, Uchio-Yamada K, Sawada K, Kunieda T, Takeuchi D (2010). Diabetes-accelerated memory dysfunction via cerebrovascular inflammation and A deposition in an Alzheimer mouse model with diabetes. Proc Natl Acad Sci.

[210] LeChatelier E, Nielsen T, Qin J, Prifti E, Hildebrand F, Falony G (2013). Richness of human gut microbiome correlates with metabolic markers. Nature..

[211] Cash JG, Kuhel DG, Basford JE, Jaeschke A, Chatterjee TK, Weintraub NL (2012). Apolipoprotein E4 impairs macrophage efferocytosis and potentiates apoptosis by accelerating endoplasmic reticulum stress. J Biol Chem.

[212] Fond AM, Ravichandran KS. Clearance of dying cells by phagocytes: mechanisms and implications for disease pathogenesis. Adv Exp Med Biol. 2016:25–49 Available from: http://link.springer.com/10.1007/978-3-319-39406-0_2. Accessed 25 Aug 2016. 10.1007/978-3-319-39406-0_2 PMC6721615 27558816

[213] Swardfager W, Lanctôt K, Rothenburg L, Wong A, Cappell J, Herrmann N (2010). A meta-analysis of cytokines in Alzheimer’s disease. Biol Psychiatry.

[214] Lai KSP, Liu CS, Rau A, Lanctôt KL, Köhler CA, Pakosh M (2017). Peripheral inflammatory markers in Alzheimer’s disease: a systematic review and meta-analysis of 175 studies. J Neurol Neurosurg Psychiatry.

[215] Holmes C, Cunningham C, Zotova E, Woolford J, Dean C, Kerr S, et al. Systemic inflammation and disease progression in Alzheimer disease. Neurology [Internet]. 2009;73:768–74. Available from: http://www.neurology.org/cgi/doi/10.1212/WNL.0b013e3181b6bb95. 10.1212/WNL.0b013e3181b6bb95 PMC2848584 19738171

[216] Sharma HS, Castellani RJ, Smith MA, Sharma A. The blood-brain barrier in Alzheimer’s disease: novel therapeutic targets and nanodrug delivery. Int Rev Neurobiol. 2012:47–90. Available from: https://linkinghub.elsevier.com/retrieve/pii/B978012386986900003X. Accessed 27 June 2012. 10.1016/B978-0-12-386986-9.00003-X 22748826

[217] Qin L, Wu X, Block ML, Liu Y, Breese GR, Hong J-S (2007). Systemic LPS causes chronic neuroinflammation and progressive neurodegeneration. Glia..

[218] Riazi K, Galic MA, Kuzmiski JB, Ho W, Sharkey KA, Pittman QJ (2008). Microglial activation and TNFα production mediate altered CNS excitability following peripheral inflammation. Proc Natl Acad Sci.

[219] Luckheeram RV, Zhou R, Verma AD, Xia B (2012). CD4 + T cells: differentiation and functions. Clin Dev Immunol.

[220] Harty JT, Tvinnereim AR, White DW (2000). CD8+ T cell effector mechanisms in resistance to infection. Annu Rev Immunol.

[221] Fehervari Z (2016). Lymphocytes in Alzheimer’s disease. Nat Immunol.

[222] Ji K, Akgul G, Wollmuth LP, Tsirka SE (2013). Microglia actively regulate the number of functional synapses. PLoS One.

[223] Krabbe G, Halle A, Matyash V, Rinnenthal JL, Eom GD, Bernhardt U (2013). Functional impairment of microglia coincides with beta-amyloid deposition in mice with Alzheimer-like pathology. PLoS One.

